# Insights into transcriptional regulation of β-D-N-acetylhexosaminidase, an N-glycan-processing enzyme involved in ripening-associated fruit softening

**DOI:** 10.1093/jxb/eru324

**Published:** 2014-08-16

**Authors:** Mohammad Irfan, Sumit Ghosh, Vinay Kumar, Niranjan Chakraborty, Subhra Chakraborty, Asis Datta

**Affiliations:** National Institute of Plant Genome Research, New Delhi 110067, India

**Keywords:** Fruit ripening, fruit ripening-specific promoter, β-Hex, RIN, SlASR1, transcriptional regulation.

## Abstract

Tomato (*Solanum lycopersicum*) fruit ripening-specific N-glycan processing enzyme, β-D-N-acetylhexosaminidase (β-Hex), plays an important role in the ripening-associated fruit-softening process. However, the regulation of fruit ripening-specific expression of *β-Hex* is not well understood. We have identified and functionally characterized the fruit ripening-specific promoter of *β-Hex* and provided insights into its transcriptional regulation during fruit ripening. Our results demonstrate that RIPENING INHIBITOR (RIN), a global fruit ripening regulator, and ABSCISIC ACID STRESS RIPENING 1 (SlASR1), a poorly characterized ripening-related protein, are the transcriptional regulators of *β-Hex*. Both RIN and SlASR1 directly bound to the *β-Hex* promoter fragments containing CArG and C_2-3_(C/G)A *cis*-acting elements, the binding sites for RIN and SlASR1, respectively. Moreover, *β-Hex* expression/promoter activity in tomato fruits was downregulated once expression of either *RIN* or *SlASR1* was suppressed; indicating that *RIN* and *SlASR1* positively regulate the transcription of *β-Hex* during fruit ripening. Interestingly, RIN could also bind to the *SlASR1* promoter, which contains several CArG *cis*-acting elements, and *SlASR1* expression was suppressed in *rin* mutant fruits, indicating that RIN also acts as a positive regulator of *SlASR1* expression during fruit ripening. Taken together, these results suggest that RIN, both directly and indirectly, through *SlASR1*, regulates the transcription of *β-Hex* during fruit ripening. The fruit ripening-specific promoter of *β-Hex* could be a useful tool in regulating gene expression during fruit ripening.

## Highlight text

Ripening-specific expression of *β-Hex*, a gene involved in the fruit-softening process, is transcriptionally regulated by the MADS-box transcription factor RIN in SlASR1-dependent and -independent manners.

## Introduction

Fleshy fruit ripening is a coordinated developmental process that imparts nutritional and sensory qualities to fruits while altering their colour, texture, aroma, and flavour (Klee and Giovannoni, 2011; Osorio *et al.*, 2013; Seymour *et al.*, 2013*a*, *b*). Therefore, understanding the regulatory mechanisms of fruit ripening may be useful in improving fruit sensory and nutritional qualities, as well as post-harvest stability. Tomato (*Solanum lycopersicum*) is a model plant for the biochemical and genetic analysis of fleshy fruit development and ripening processes. Previous studies on tomato have provided insights into N-glycans that are present in the pericarp of fruits and accumulate during the ripening process (Priem and Gross, 1992; Priem *et al.*, 1993), and how the N-glycosylation process influences fruit ripening (Handa *et al.*, 1985; Meli *et al.*, 2010). The ripening-stimulating activity of free N-glycans has previously been noted by injecting them into mature green tomato fruits, which resulted in induction of red colouration and ethylene production (Priem and Gross, 1992). Moreover, blocking of N-glycosylation delayed fruit ripening (Handa *et al.*, 1985). In this context, the role of a particular enzyme during the ripening process was elucidated by molecular and biochemical characterization of the fruits either suppressing or overexpressing the ripening-specific N-glycan-processing enzymes β-D-N-acetylhexosaminidase (β-Hex) and α-mannosidase (Meli *et al.*, 2010; Ghosh *et al.*, 2011).

β-Hex is a member of the glycosyl hydrolase family 20 (GH20). The homologues of this enzyme are present in a wide range of organisms from prokaryotes to eukaryotes (Slamova *et al.*, 2010). GH20 family members catalyse cleavage of the terminal N-acetyl-β-D-glucosamine (β-D-GlcNAc) and N-acetyl-β-D-galactosamine (β-D-GalNAc) residues present in N-acetyl-β-D-hexosaminides. In plants, β-Hex-mediated removal of the β-D-GlcNAc residues from N-glycans results in generation of the paucimannosidic N-glycans that are present in most plant glycoproteins (Gutternigg *et al.*, 2007; Strasser *et al.*, 2007; Liebminger *et al.*, 2011). β-Hex activity increases during ripening of many fruits (Jagadeesh and Prabha, 2002; Jagadeesh *et al.*, 2004) and RNA interference (RNAi)-mediated suppression of *β-Hex* expression resulted in a reduced rate of fruit softening in climacteric tomato and non-climacteric capsicum (*Capsicum annuum*) (Meli *et al.*, 2010; Ghosh *et al.*, 2011). Transgenic analysis revealed that β-Hex-mediated trimming of N-glycans attached to cell wall glycoproteins is a regulatory mechanism in controlling the fruit softening process (Meli *et al.*, 2010). Interestingly, suppression of *β-Hex* expression resulted in reduced transcript levels of the ethylene response factor (ERF) family transcription factor and several ripening-related enzymes involved in degradation of the cell wall cellulose, hemicellulose, and pectin polysaccharides. Besides, ethylene positively regulates *β-Hex* and its expression is suppressed in ripening-impaired mutants such as *ripening-inhibitor* (*rin*), *non-ripening* (*nor*), and *never-ripe* (*Nr*), which are deficient in ripening-associated ethylene biosynthesis or ethylene perception (Tigchelaar *et al.*, 1978; Wilkinson *et al.*, 1995; Vrebalov *et al*., 2002; Meli *et al.*, 2010). Moreover, the involvement of *β-Hex* in ripening-associated softening of peach fruit (*Prunus persica*) has recently been reported (Cao *et al.*, 2014). Inhibition of β-Hex activity in peach fruits resulted in delayed fruit softening, a reduced level of ethylene production, and reduction in the transcript level of *β-Hex* as well as genes involved in ethylene biosynthesis and cell wall degradation (Cao *et al.*, 2014). Therefore, downregulation of ethylene and cell wall degradation-related transcripts in *β-Hex*-suppressed fruits suggested the existence of a feedback mechanism in the fruit-softening process.

Genetic and biochemical analysis of the tomato MADS box transcription factor RIPENING INHIBITOR (RIN) provided key insights into transcriptional control of the fruit ripening process (Vrebalov *et al.*, 2002; Giovannoni, 2007; Fujisawa *et al.*, 2013; Zhong *et al.*, 2013). RIN is considered a master regulator of ripening, since mutation hindered almost the entire fruit-ripening process, including colour development, softening, and the climacteric rise in respiration rate and ethylene production (Vrebalov *et al.*, 2002). The RIN-mediated transcriptional regulation of fruit ripening has been well studied using chromatin immunoprecipitation and transcriptome and proteome analyses, which revealed several direct and indirect RIN target genes (Ito *et al.*, 2008; Fujisawa *et al.*, 2011; Martel *et al.*, 2011; Fujisawa *et al.*, 2012; Kumar *et al.*, 2012; Qin *et al.*, 2012; Fujisawa *et al.*, 2013; Zhong *et al.*, 2013). These included ripening-related genes involved in the ethylene biosynthesis and signalling pathway, cell wall modification, and carotenoid and aroma biosynthesis, and also some key transcriptional regulators such as NON-RIPENING, COLORLESS NON-RIPENING, and FRUITFULL1. Taken together, these studies suggested that RIN constitutes an upstream component of the ethylene-dependent and -independent ripening pathways.

In both tomato and capsicum fruits, *β-Hex* transcripts showed ripening-specific accumulation that can be correlated with the increase in protein level and enzyme activity during fruit ripening (Meli *et al.*, 2010; Ghosh *et al.*, 2011). This temporal relationship between the transcript and protein levels with the enzyme activity indicated that the rate of *β-Hex* transcription might be the controlling factor in determining its protein level and enzyme activity during ripening. However, how *β-Hex* transcription is regulated during fruit ripening is currently unknown. Therefore, we have identified and functionally characterized the fruit ripening-specific promoter of *β-Hex* to understand how its transcription is regulated during fruit ripening. The results of the present study demonstrate that RIN acts as a positive transcriptional regulator of *β-Hex*. In *rin* fruits, the transcript level of *β-Hex* was downregulated and *β-Hex* promoter-driven expression of *GUS* reporter was significantly reduced. Moreover, DNA–protein interaction analysis by electrophoretic mobility shift assay (EMSA) confirmed binding of RIN to the *β-Hex* promoter sequence. Further, yeast one-hybrid (Y1H) screening and EMSA analysis led to the identification of ABSCISIC ACID STRESS RIPENING 1 (SlASR1) as another *β-Hex* promoter-interacting protein. Virus-induced gene silencing (VIGS)-mediated suppression of *SlASR1* in fruits caused transcriptional downregulation of *β-Hex*. However, the transcript level of *SlASR1* was upregulated during ripening of wild-type tomato and inhibited in the *rin* mutant. Moreover, RIN also interacted with the *SlASR1* promoter in EMSA. Thus, *RIN* could both directly and through *SlASR1* regulate the expression of *β-Hex* during fruit ripening.

## Materials and methods

### Plant materials and growth conditions

Tomato (cv. Pusa Ruby) and capsicum (cv. California Wonder) seeds were obtained from the National Seeds Corporation Ltd, New Delhi. Tomato mutants used in the study were procured from the Tomato Genetics Resource Center, University of California at Davis and were in an Ailsa Craig background. Seeds were germinated in pre-sterilized soil and later transplanted into pots containing soil, agropeat and vermiculite (2:1:1). Plants were grown in a growth chamber with 25/22°C day/night temperature, 65% relative humidity and 16/8h light/dark regime. For the analysis, fruits were harvested at 3, 5, 10, 15, and 20 days after anthesis (DAA) and at the mature green (MG), breaker (BR), pink (P), and red ripe (RR) stages after tagging the flowers at anthesis. Fruits after ~40 days of anthesis were considered MG (the surface of the tomato was completely green; the shade of colour varied from light to dark), MG + 4 days as BR stage, B + 2 days as P stage, and P + 3 days as RR stage.

### Isolation and analysis of *β-Hex* promoter

*β-Hex* promoters from tomato and capsicum were isolated using the Universal GenomeWalker^TM^ Kit (Clontech, USA). Genomic DNA was extracted from leaves following the cetyl trimethyl ammonium bromide (CTAB) method (Doyle and Doyle, 1987), and digested separately with PvuII, XmnI, MscI, DraI, and SspI enzymes, providing five genome walking libraries. In capsicum, instead of PvuII, SmaI was used. PCR was carried out separately for each library with a GenomeWalker adapter-specific primer (AP1) and a gene-specific primer (GSP1). Primary PCR product was used as a template to perform nested amplification using AP2 and GSP2 primers. The amplified PCR product was cloned into pGEM-T Easy vector and sequenced. Tomato and capsicum *β-Hex* promoter sequences (GenBank accession nos. KJ494862 and KJ494863, respectively) were analysed *in silico* to find out putative *cis*-acting elements using NewPLACE (Higo *et al.*, 1999), PlantCARE (Lescot *et al.*, 2002), and MatInspector (Cartharius *et al.*, 2005) servers, and the FUZZNUC program (EMBOSS package; Rice *et al.*, 2000).

### Construction of promoter::*GUS* fusion

Tomato and capsicum *β-Hex* promoter::*GUS* fusion constructs were prepared in binary vector pBI121 after replacing the CaMV 35S promoter with the *β-Hex* promoter. Tomato and capsicum *β-Hex* promoters were PCR amplified using high fidelity *pfx* DNA polymerase to incorporate appropriate restriction sites and cloned into pBI121 following standard restriction digestion and ligation methods. Positive clones were transformed into *Agrobacterium tumefaciens* (strain EHA105) following electroporation.

### *Agrobacterium*-based transient and stable transformation

Agroinjection was performed into the pericarp of tomato fruits at MG stage, as described previously (Orzaez *et al.*, 2006) but with some modifications. Primary culture was raised at 28°C in YEP medium containing rifampicin (50mg ml^–1^) and kanamycin (100mg ml^–1^) using a single isolated colony of *Agrobacterium* transformed with an appropriate construct. Further, a part of the culture (200 μl) was used to inoculate 50ml induction medium (0.5% beef extract, 0.1% yeast extract, 0.5% peptone, 0.5% sucrose, 2mM MgSO_4_, 20mM acetosyringone, and 10mM MES, pH 5.6) with antibiotics and grown at 28°C until the optical density at 600nm (OD600) attained 0.8–1.0. Cells were then recovered and resuspended in 50ml of infiltration medium (10mM MgCl_2_, 10mM MES, and 200mM acetosyringone, pH 5.6) and further incubated at room temperature with gentle agitation (20rpm) for 2h. Culture was then injected into the fruits at 4–5 spots with the help of a syringe (1ml; needle size, 0.33313mm). The needle was introduced up to 3–4mm in depth into the fruit tissue and infiltration solution was gently injected.

To generate transgenic tomato plants, cotyledons from 2-week-old seedlings were used as described previously (Fillati *et al.*, 1987) but with some modifications. Briefly, tomato seeds were sterilized using 4% commercial bleach and germinated on Murashige and Skoog (MS) medium. After 2 weeks of germination, cotyledons were cut and co-cultivated for 30min with *Agrobacterium* in MS medium containing acetosyringone (0.1 μM). Cotyledons were then collected for selection on MS plates containing kanamycin (50mg l^–1^), cefotaxime (250mg l^–1^), and zeatine (1ng l^–1^). When plantlets regenerated, they were transferred to rooting medium [MS containing kanamycin (50mg l^–1^)], cefotaxime (250mg l^–1^), and IAA (1ng l^–1^)]. Transgenic seeds were germinated in MS medium containing kanamycin (50mg l^–1^) to get progeny plants.

### RNA isolation and quantitative RT-PCR

RNA was isolated (Menke *et al.*, 1999) and purified using an RNeasy Mini Kit (Qiagen). Five micrograms of total RNA were quantified using a nanodrop (ND 1000) and reverse transcribed to cDNA using superscript II RT (Invitrogen). qRT-PCR was performed using One Step Real Time RT-PCR (Applied Biosystems) with SYBR Green, as described previously (Ghosh *et al.*, 2013). The analysis was done in triplicate from cDNA derived from at least two independent experiments. Using the 2^–ΔΔ CT^ method (Bovy *et al.*, 2002), data are presented as the fold change in gene expression or percentage of expression, normalized to the endogenous reference gene (*actin*) and relative to control. The oligonucleotide primers used for qRT-PCR are listed in Supplementary Table S1 (at *JXB* online).

### GUS histochemical and fluorometric assay

The GUS assays were performed as described previously (Jefferson *et al.*, 1987) but with some modifications. For histochemical GUS analysis, 20-day-old seedlings, roots, leaves, flowers, and fruits at different stages of development and ripening were collected from wild-type and promoter::*GUS* fusion transgenic plants. The seedlings, roots, leaves, and flowers were immersed whole in GUS-staining solution (100mM sodium phosphate buffer, pH 7.0, 10mM EDTA, 0.5mM K_3_Fe(CN)_6_, 0.5mM K_4_Fe(CN)_6_.3H_2_O, 0.1% Triton-X100, 20% methanol, and 1mM X-Gluc), while fruits were cut into transverse sections, dipped in GUS-staining solution, vacuum infiltrated, and incubated overnight at 37°C in darkness followed by destaining in 75% ethanol; they were photographed using a Canon G6 powershot with 4X zoom or Canon EOS 400D DIGITAL (10.1 megapixel) and Nikon AZ100 5X microscope.

Quantitative GUS activity was determined by measuring the production of 4-methylumbelliferone (4-MU) as pmol 4-MU mg^–1^min^–1^. For this, samples from three individual plants of single copy transgenic lines were pooled and homogenized in 400 μl GUS extraction buffer (50mM sodium phosphate buffer, pH 7.0, 10mM DTT, 10mM EDTA, 0.1% sodium lauryl sarcosine, and 0.1% Triton X100); supernatant was collected after centrifugation. Further, 50 μl of supernatant was added to 450 μl MUG assay buffer (GUS extraction buffer containing 10mM MUG) and incubated at 37 °C. After 1h incubation, 100 μl of aliquots were removed and mixed with 900 μl 0.2M Na_2_CO_3_ to terminate the reaction. GUS activity was measured using a fluorometer (Cary Eclipse, Varian) with excitation at 380nm and emission at 454nm.

### Y1H assay

Y1H assay was carried out using a Matchmaker One-Hybrid Library Construction and Screening Kit (Clontech). Tomato *β-Hex* promoter sequence was PCR amplified to append an EcoRI site at the 5′-end and a SpeI site at the 3′-end and cloned upstream of the *His3* reporter in pHIS2.1 vector to make bait construct (pHIS2.1-HP). For cDNA preparation, total RNA was isolated from P stage tomato fruits and enriched for mRNA (Dynabeads® mRNA Purification Kit, Invitrogen) and first-strand cDNA synthesis was performed following the manufacturer’s protocol. First-strand cDNA thus generated was amplified using long-distance (LD)-PCR. Further, ds cDNA generated by LD-PCR was column purified using a CHROMA SPIN TE-400 column. pHIS 2.1/bait plasmid was transformed into competent yeast Y187 cells and transformed yeasts were selected on SD/-Trp medium. To suppress the basal level expression of *His3* from the reporter-bait plasmid 3-AT (3-amino-1,2,4-triazole) was used. Competent cells were prepared from bait plasmid-transformed yeast cells and used for library screening. The transformed cells were spread on SD/-Leu/-Trp/-His/+ 5mM 3-AT (TDO + 3-AT) plates. Yeast colonies, thus obtained, were streaked three times on TDO + 3-AT to segregate false prey plasmids and eliminate false positives. To identify the promoter-interacting proteins, prey plasmids were rescued from yeast cells and sequenced. To validate the interaction, rescued prey plasmids were co-transformed with pHIS2.1-HP into Y187 cells and selected on TDO + 3-AT media.

### EMSA

EMSA was performed as described previously (Shkolnik and Bar-Zvi, 2008) with minor modifications. Briefly, a 200bp *β-Hex* promoter fragment upstream of ATG was PCR amplified using primers listed in Supplementary Table S1. HindIII/XbaI-digested *β-Hex* promoter fragment was end filled with [α-P^32^]CTP (3000 Ci mmol^–1^, 50 µCi), using DNA polymerase I (Klenow) fragment (New England Biolabs) and purified using a Sephadex G-50 column. EMSA was performed with [α-P^32^]CTP labelled *β-Hex* promoter fragments incubated with purified GST-SlASR1 protein in gel-shift assay binding buffer (20mM HEPES, pH 7.5, 20% glycerol, 0.05 μg poly(dIdC):poly(dIdC), 0.8mM ZnCl_2_, 2.5mM EDTA, 2.5mM DTT, and 25mM NaCl) at 25 °C for 30min. For competition assays, unlabelled promoter fragments were used as specific competitive inhibitors and an unrelated DNA [200bp region downstream of ATG of tomato *actin* (FJ532351.1)] was used as a non-specific competitor. After incubation for 30min, the reaction was loaded onto 6% native PAGE. The gel was run at room temperature at a constant current of 10 mA using 0.5X TBE running buffer. The protein–DNA complexes and free probes were visualized by autoradiography.

*In vitro* binding of RIN protein (AF448522.1) with the CArG box present within the promoters of *β-Hex* and *SlASR1* (obtained from the solgenomics tomato genome database) was carried out with both normal and mutated double-strand radiolabelled probe containing the CArG box and its flanking sequences. 5′-end radiolabelling of probes was carried out with T4 polynucleotide kinase enzyme using [γ-P^32^]ATP (3000 Ci mmol^–1^, 50 µCi). The RIN protein was incubated with radiolabelled probe in gel-shift assay binding buffer (20mM HEPES, pH 7.5, 20% glycerol, 0.05 μg poly(dIdC):poly(dIdC), 10mM MgCl_2_, 2.5mM EDTA, 2.5mM DTT, and 25mM NaCl) for 30min at 25 °C. The assay was also performed using a 200-bp promoter fragment of *β-Hex* and a 142-bp region of the *SlASR1* promoter containing the CArG box. The specificity of binding was further determined using cold competitor DNAs (unlabelled probe).

### VIGS

pTRV1 and pTRV2 were obtained from the Arabidopsis Biological Resource Center (Columbus, OH, USA) and used as VIGS vectors for silencing of the *SlASR1* gene as described previously (Liu *et al.*, 2002). In brief, 330bp coding sequence of *SlASR1* (Solyc04g071610.2.1) was PCR amplified using Advantage 2 DNA polymerase (Clontech) and cloned into pTRV2 at the EcoRI/XbaI site. The empty pTRV2 and *SlPDS* cloned into pTRV2 (pTRV2-*SlPDS*) were used as controls. All the vectors (pTRV1, pTRV2, pTRV2-*SlPDS*, and pTRV2-*SlASR1*) were mobilized into *Agrobacterium* (strain GV3101). A 50ml culture of *Agrobacterium* was grown separately for different vectors as described earlier for *Agrobacterium*-based transient assay. For agroinjection, a mixture of *Agrobacterium* suspension containing pTRV1 and pTRV2 vectors at a ration of 1:1 were prepared. Fruits at 30 DAA were injected with 1ml *Agrobacterium* culture through the carpopodium with the help of a syringe to initiate VIGS.

### ACC and ABA treatment

Tomato seeds were germinated on MS media. 15-day-old seedlings were transferred to liquid MS medium containing 1mM ACC or 0.1mM ABA, harvested at different time points, and frozen immediately in liquid nitrogen. Seedlings transferred to MS liquid medium without ACC/ABA were used as a control.

### Statistical analysis

All values are presented as the means (± SE) of biological replicates. Three technical replicates were considered for each biological sample and used to calculate one average value per biological sample.

## Results

### Isolation and transgenic analysis of *β-Hex* promoter

A comparison of the genomic (EU244856) and cDNA (EU244854) sequences revealed that tomato *β-Hex* gene consists of two exons (778bp and 950bp, respectively) that are interrupted by a 782-bp intron (Supplementary Figure S1 at *JXB* online). To isolate the promoter sequence, directional genome walking PCR was carried out using a set of adapter and gene-specific primers that were designed based on the genomic sequence (Supplementary Figure S2 at *JXB* online). Comparison of the sequences obtained from the genome walking PCR with the *β-Hex* gene revealed a 1001-bp sequence (KJ494862) upstream of the translation start site. Following random amplification of cDNA ends (5′-RACE, Clontech), the putative transcription start site (TSS) was determined to be 9bp upstream of the translation start site (Supplementary Figure S1). All the sequences were verified with the recently published tomato genome sequence (solgenomics.net).

**Fig. 1.**
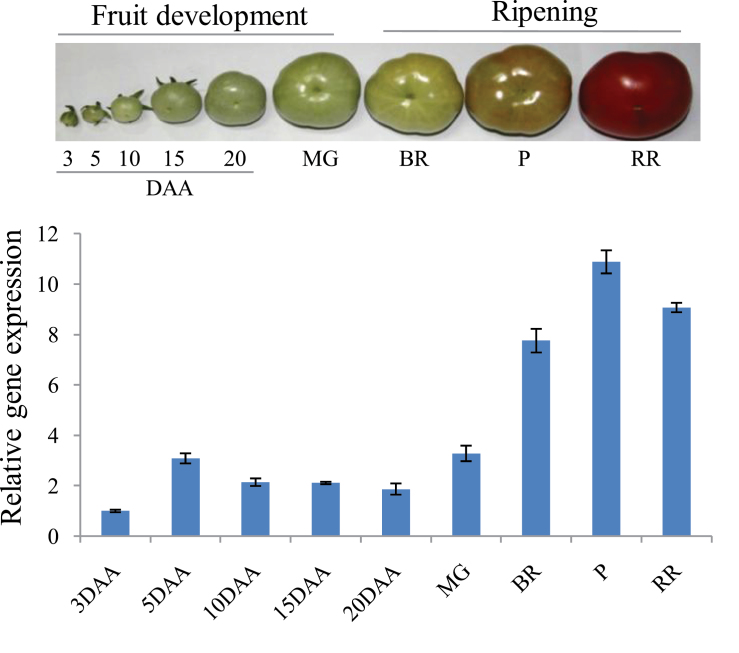
Expression profiles of *β-Hex* during tomato fruit development and ripening stages. Flowers were tagged at anthesis and fruits were harvested at 3 to 20 DAA and MG, BR, P, and RR stages. Transcript level of *β-Hex* was measured by qRT-PCR analysis using tomato actin as an endogenous control. Data are presented as the mean (±SE) of at least three biological replicates.

**Fig. 2.**
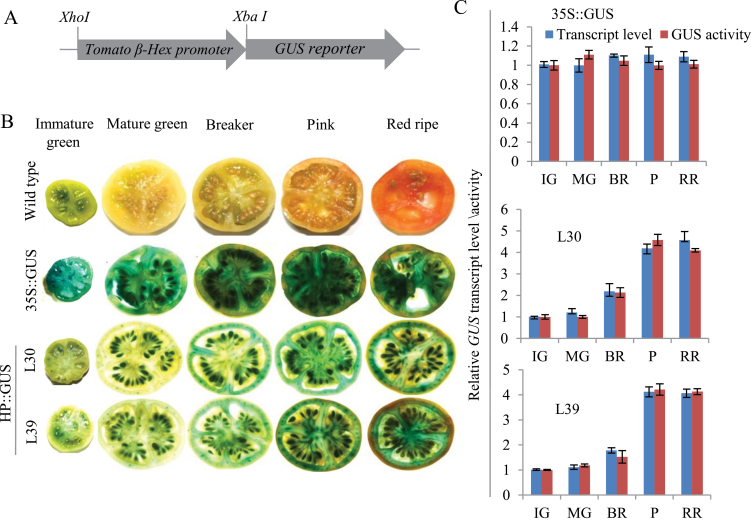
Determination of *GUS* transcript level and activity (histochemical and fluorometric assays) in HP::GUS and CaMV 35S::GUS tomato fruits. (A) Schematic representation of HP::GUS fusion construct (*GUS* gene fused with tomato *β-Hex* promoter). (B) Comparative analysis of histochemical GUS staining of fruits harvested from the wild type untransformed plants and tomato transgenic plants transformed with HP::GUS (*GUS* under the control of tomato *β-Hex* promoter) and 35S::GUS (*GUS* under the control of CaMV 35S) constructs. L30 and L39 denote two independent tomato transgenic lines generated by transforming with HP::GUS construct. (C) *GUS* transcript level and activity in transgenic fruits were determined by qRT-PCR and fluorometric GUS assay, respectively. Data are presented as the mean (±SE) of two biological replicates. Immature green (IG) represents 15 DAA fruits.

**Fig. 3.**
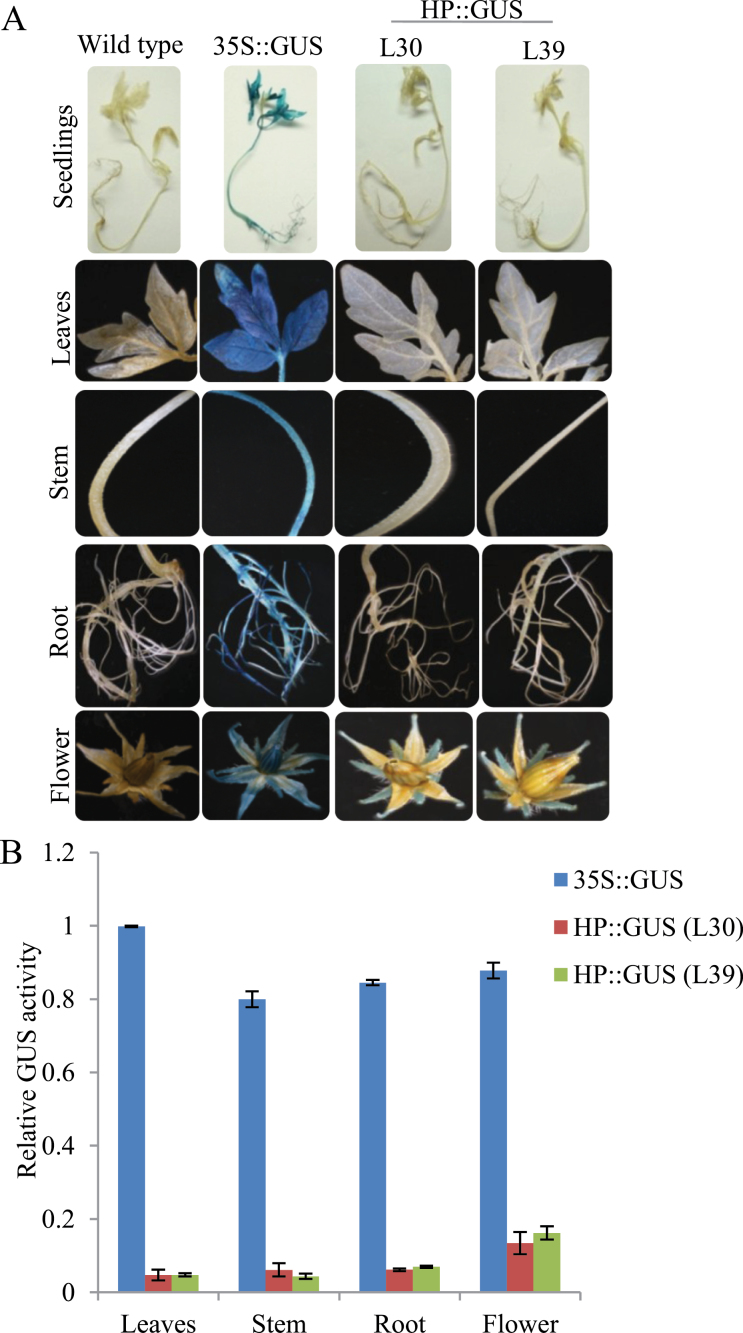
Comparative analysis of GUS activity in different plant parts through histochemical and fluorometric assays. (A, B) Seedlings (20 days old), leaves, stems, roots, and flowers of wild type (cv. Pusa Ruby) and transgenics were used for the histochemical (A) and fluorometric (B) assays. Data are presented as the mean (±SE) of two biological replicates.

**Fig. 4.**
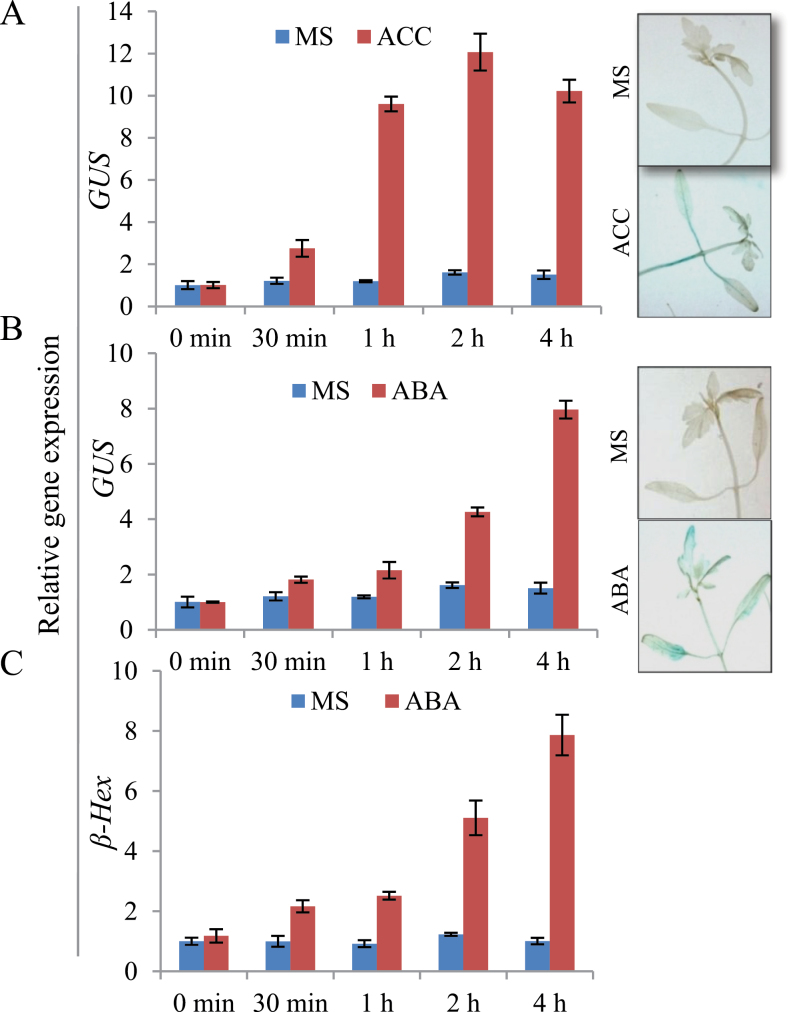
Activation of the *β-Hex* promoter in response to ethylene and ABA. Seedlings were treated with 1mM ACC or 0.1mM ABA in MS medium. *GUS* transcript level and activity were determined by qRT-PCR analysis and histochemical GUS assay, respectively. Histochemical assay was carried out after 4h of treatment. (A) *GUS* transcript level and activity were determined in HP::GUS (L30) seedlings after ACC treatment. (B) *GUS* transcript level and activity were determined in HP::GUS (L30) seedlings after ABA treatment. (C) *β-Hex* transcript level was determined in wild-type seedlings after ABA treatment. Data are presented as the mean (±SE) of atleast three biological replicates.

Through northern hybridization, *β-Hex* transcript level was found to accumulate maximally during tomato ripening with a peak at the P stage (Meli *et al.*, 2010). In order to corroborate the earlier results, quantitative RT-PCR (qRT-PCR) analysis was carried out to examine the transcript level of *β-Hex* throughout fruit development and ripening of tomato. The analysis revealed maximum transcript levels at the P stage of ripening as compared to the developing and unripe fruits (Fig. 1). Further, to investigate the fruit-ripening-specific activation of the promoter, tomato transgenic plants were generated by constructing tomato *β-Hex* promoter::*β-glucuronidase* (HP::GUS) gene fusions (Figs 2A–C, and 3A and B). We included the 1001-bp sequence upstream of the translation start site as the tomato *β-Hex* promoter. 25 independent transgenic lines were generated for the HP::GUS construct and 10 lines were initially analysed by histochemical GUS staining to determine the ripening-specificity of the promoter. Finally, two independent T2 transgenic lines (L30 and L39) were selected for detailed examination of the tissue and organ-specific activation of the *β-Hex* promoter. For this, fruits of different developmental and ripening stages and roots, stems, leaves, and flowers were taken for histochemical and fluorometric GUS activity assays and for determining *GUS* transcript levels (Figs 2B–C, and 3A and B). The analysis revealed maximum transcript levels and activity of GUS in ripening fruit, quite similar to the *β-Hex* expression pattern during tomato ripening (Fig. 1). Consistent with the role of *β-Hex* in fruit pericarp softening, prominent GUS activity was recorded in the fruit pericarp tissue (Fig. 2B). However, GUS activity was not detected in seedlings, roots, stems, or leaves. In the case of flowers, slight GUS activity was noticed in sepals; however, no GUS staining was observed in petals (Fig. 3A and B). In contrast, constitutive expression of *GUS* was noticed in seedlings, roots, stems, leaves, flowers, and fruits when the gene was expressed under the control of the CaMV 35S promoter. *β-Hex* expression is regulated by the plant hormone ethylene, which plays a pivotal role in tomato ripening (Meli *et al.*, 2010). Therefore, the transcript level and activity of GUS was determined after treatment of HP::GUS transgenic seedlings with 1-aminocyclopropane-1-carboxylic acid (ACC), the precursor of ethylene. The upregulation of *GUS* transcript and activity indicated the activation of the *β-Hex* promoter in response to ethylene (Fig. 4A). Taken together, these results substantiate the fruit ripening-specific and ethylene-inducible expression of *β-Hex* which was undetectable in other plant parts (Meli *et al.*, 2010). These analyses also suggest that the 1001-bp upstream sequence of *β-Hex* does contain the *cis*-acting elements involved in fruit-ripening specific expression of the gene, and thus can be regarded as the full-length *β-Hex* promoter.

In order to get an overview of the putative *cis*-acting regulatory elements that may control the transcription of *β-Hex*, the promoter sequence was analysed through NewPLACE (https://sogo.dna.affrc.go.jp), PlantCARE (bioinformatics.psb.ugent.be), and MatInspector (www.genomatix.de) for the identification of the putative transcription factor binding sites. The analysis revealed an array of *cis*-acting elements that can be recognized by the transcription factors involved in plant hormone signalling such as ethylene, auxin, gibberellin, abscisic acid, and brassinosteroids (Supplementary Table S2 at *JXB* online). Crosstalk of these hormones in fruit ripening is discussed elsewhere (Osorio *et al.*, 2013; Seymour *et al.*, 2013a). Among the *cis*-acting elements, MADS-box transcription factor RIN binding sites (CArG box; Fujisawa *et al.*, 2013) were of particular interest, because *β-Hex* expression was downregulated in *rin* mutant fruit (Fig. 6D; Meli *et al.*, 2010).

**Fig. 5.**
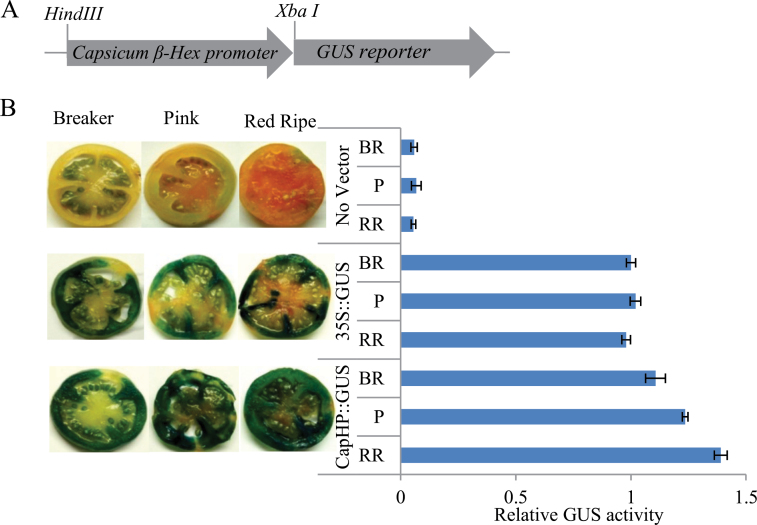
Activation of capsicum *β-Hex* promoter (CapHP) in tomato fruit. MG tomato fruits were agroinjected with the promoter::GUS constructs. Agroinjected fruits were harvested at BR, P, and RR stages of ripening, and histochemical and fluorometric GUS assays were performed. (A) Schematic representation of capsicum *β-Hex* promoter::GUS fusion construct (CapHP::GUS). (B) Histochemical and fluorometric GUS assays of tomato fruits agroinjected with *Agrobacterium* strains carrying 35S::GUS or CapHP::GUS constructs. *Agrobacterium* strain without any vector was used as a control. Data are presented as the mean (±SE) of two biological replicates.

*β-Hex* exhibits similar expression profiles during the ripening of both climacteric tomato (Meli *et al.*, 2010) and non-climacteric capsicum (Ghosh *et al.*, 2011). This prompted us to test activation of the capsicum *β-Hex* promoter (KJ494863) in tomato fruit. Capsicum *β-Hex* promoter sequence (951bp) was isolated following the directional genome walking PCR approach and used to drive the expression of the *GUS* reporter (Fig. 5A and Supplementary Figure S3, at *JXB* online). Functional analysis of the capsicum *β-Hex* promoter was carried out by *Agrobacterium*-mediated transient expression (agroinjection) in tomato fruits as described in the materials and methods section. Fruits were agroinjected at the MG stage and harvested at BR, P, and RR stages for measuring GUS activity by histochemical and fluorometric assays (Fig. 5B). Capsicum *β-Hex* promoter-driven expression of *GUS* in tomato fruit indicated activation of the capsicum promoter in tomato fruit. This result suggests the existence of a conserved transcriptional mechanism for the expression of *β-Hex* in these *Solanaceae* fruits which have different ripening behaviour. *In silico* analysis of the capsicum and tomato *β-Hex* promoters identified several conserved *cis*-acting elements, including CArG boxes that may be involved in controlling the transcription of *β-Hex* in both climacteric and non-climacteric fruits (Supplementary Table S2).

### Transcriptional regulation of *β-Hex* by RIN

**Fig. 6.**
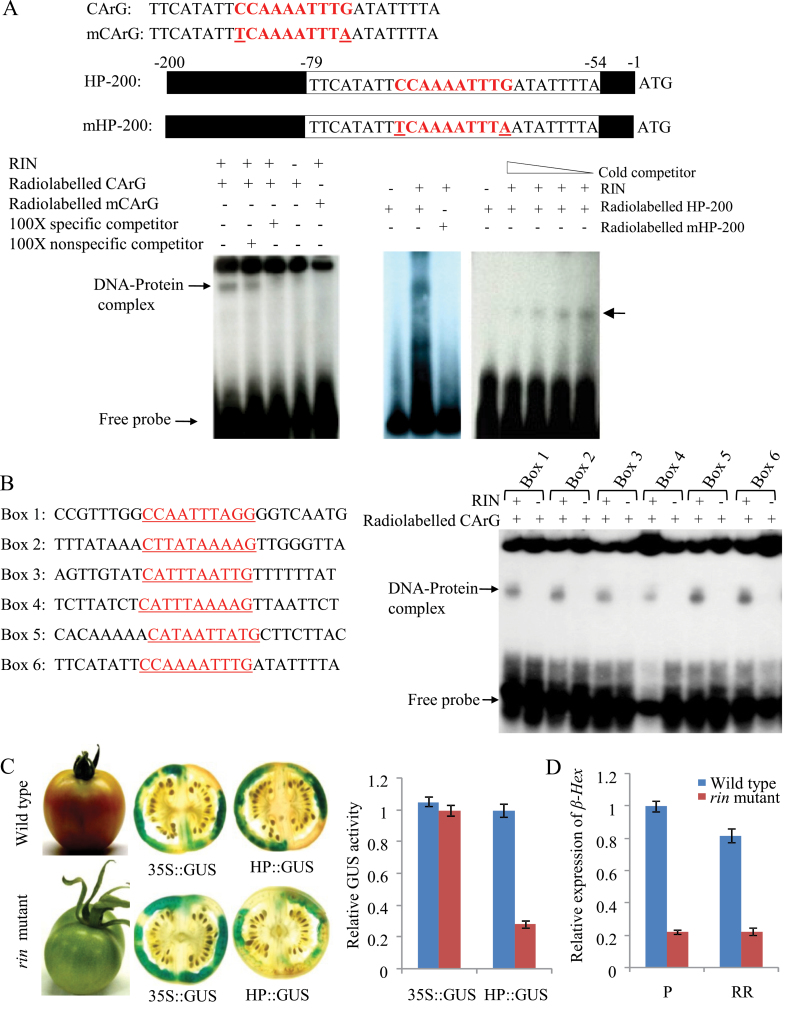
RIN-mediated transcriptional regulation of *β-Hex.* (A) EMSA demonstrates the formation of DNA–protein complex between RIN and tomato *β-Hex* promoter fragments. EMSA was performed using double-stranded radiolabelled probes containing either 26-bp or 200-bp promoter fragments with normal (CArG and HP-200) or mutated (mCArG and mHP-200) CArG box elements with their flanking sequences. Cytosine (C) and guanine (G) were replaced with thymine (T) and adenine (A), respectively, to create mutated CArG probes. Specificity of the DNA–protein complex was analysed by using competitor DNAs. Arrows indicate DNA–protein complex. (B) EMSA shows binding of RIN protein to six different CArG boxes identified within the tomato *β-Hex* promoter. (C) Activation of the *β-Hex* promoter in the wild type (cv. Ailsa Craig) and *rin* mutant was determined by transient expression of *GUS* reporter in fruits. Mature green tomato fruits were agroinjected with HP::GUS construct. Agroinjected fruits were harvested at R stage and histochemical and fluorometric GUS assays were performed. Control fruits were agroinjected with 35S::GUS construct for constitutive expression of GUS reporter driven by CaMV 35S promoter. (D) Relative expression of *β-Hex* in wild type (cv. Ailsa Craig) and *rin* mutant fruits was determined by qRT-PCR using actin as an endogenous control. Data are presented as the mean (±SE) of two biological replicates.

The MADS-box transcription factor RIN modulates gene expression during fruit ripening by binding to the CArG box [C(T/A/C)(A/T)_6_(A/T/G)G] present within the promoter region of the fruit ripening-related genes (Ito *et al.*, 2008; Martel *et al.*, 2011; Fujisawa *et al.*, 2013). *In silico* analysis revealed six CArG boxes within the *β-Hex* promoter sequence (Supplementary Figure S4 at *JXB* online). Moreover, *β-Hex* expression was suppressed up to 80% in *rin* mutants as compared to wild-type fruit (Fig. 6D; Meli *et al.*, 2010). These results suggest RIN-mediated transcriptional control of *β-Hex* expression during fruit ripening. To substantiate these results and test direct regulation by RIN, the ability of RIN protein to bind the *β-Hex* promoter was determined by EMSA (Fig. 6A, B). For this, the N-terminal region (1–139aa) of RIN (AF448522.1), including the DNA-binding domain and dimerization interface (CD search, http://www.ncbi.nlm.nih.gov/Structure/cdd/wrpsb.cgi), was expressed in *E. coli* and purified (Supplementary Figure S5B, at *JXB* online), given that a C-terminal truncated version of RIN produced clear signal as compare to the full-length protein in EMSA (Ito *et al.*, 2008). For the DNA bait, we initially used two *β-Hex* promoter fragments of different lengths (26 and 200bp) containing the same CArG box (Box 6), present at –62bp to –71bp, close to the TSS in comparison to the other five CArG boxes (Fig. 6A; Supplementary Figure S4). In EMSA, DNA–protein complex was detected when wild-type CArG box was used. However, DNA–protein complex was not formed in the case of mutated CArG box (C and G were replaced with T and A, respectively). Moreover, the specificity of the DNA–protein complex was determined by using unlabelled specific (CArG probe sequence) and non-specific (tomato *actin* sequence) competitor DNAs (Fig. 6A). These results suggest that RIN specifically interacts with the CArG box sequence present within the *β-Hex* promoter. We next tested whether RIN could also interact with five additional CArG boxes identified within the *β-Hex* promoter sequence and whether these six CArG boxes have different binding specificities with the RIN protein (Fig 6B and Supplementary Figure S4). For this, 26-bp *β-Hex* promoter fragments including the CArG box along with their flanking sequences were used as DNA baits. Although DNA–protein complexes were noticed for all the six CArG boxes, EMSA gave intense signals in CArG boxes (box 5 and 6) close to the TSS; suggesting efficient binding of RIN to these two CArG boxes compared to the other four boxes. In order to further confirm the transcriptional regulation of *β-Hex* expression by RIN, *β-Hex* promoter-driven expression of *GUS* was compared in wild-type and *rin* mutant fruits, following *Agrobacterium*-mediated transient expression (Fig. 6C). Histochemical staining and fluorometric assay revealed significantly reduced activity of GUS in *rin* mutants as compared to wild-type fruits when *GUS* expression was driven by the *β-Hex* promoter. In contrast, similar levels of GUS activity were detected in wild-type and *rin* mutant fruits under the control of the CaMV 35S constitutive promoter. Altogether, these results demonstrate that the transcription factor RIN positively regulates the expression of *β-Hex*.

### Y1H assay identified SlASR1 as a *β-Hex* promoter interacting protein

Besides the CArG box, other putative transcription factor binding sites were also identified within the *β-Hex* promoter (Supplementary Table S2), suggesting that, in addition to RIN, *β-Hex* expression might be regulated by other transcription factors as well. To test this possibility, *β-Hex* promoter-interacting proteins were identified by Y1H screening using a cDNA library generated from PolyA+ RNAs isolated from P stage tomatoes as described in the materials and methods section. Y1H screening revealed ABSCISIC ACID STRESS RIPENING 1 (SlASR1; Solyc04g071610.2.1), ERF6 (Solyc01g065980.2.1), calmodulin (Solyc02g094000.1.1), and a putative calmodulin-binding protein (Solyc10g081040.1.1) as the candidate transcriptional regulators of *β-Hex*. In order to test whether these promoter-interacting proteins are expressed during fruit ripening, their transcripts levels were determined during tomato ripening. qRT-PCR expression analysis revealed increased transcript accumulation for these promoter-interacting proteins during tomato ripening (Fig. 8A and Supplementary Figure S6). The expression patterns of these promoter-interacting proteins were quite similar to *β-Hex* expression during fruit ripening (Fig. 1; Meli *et al.*, 2010), suggesting their involvement in transcriptional regulation of *β-Hex*. We selected SlASR1 for further characterization because three putative SlASR1-binding sites [C_2–3_(C/G)A, Kalifa *et al.*, 2004a; Shkolnik and Bar-Zvi, 2008] could be recognized within the *β-Hex* promoter region. ASR1-family proteins probably have dual functions, as chaperones in cytosol and transcriptional regulators in the nucleus (Kalifa *et al.*, 2004a; Ricardi *et al.*, 2012). Although the exact physiological role of *SlASR1* was not elucidated, *SlASR1* abolished abscisic acid (ABA) and glucose responses, and improved stress tolerance when overexpressed in *Arabidopsis* and tobacco, respectively (Kalifa *et al.*, 2004b; Shkolnik and Bar-Zvi, 2008). In grapefruit, an ASR1 homologue (VvMSA) was proposed to be involved in sugar and ABA signalling (Cakir *et al.*, 2003). Recently, candidate transcriptional targets of *SlASR1* under water-stressed conditions, including cell wall-related and aquaporin genes, were identified (Ricardi *et al.*, 2014). The transcript level of *SlASR1* was upregulated during tomato ripening (Fig. 8A and Iusem *et al.*, 1993); however, its role in fruit ripening is yet to be examined.

### Transcriptional regulation of *β-Hex* by SlASR1

**Fig. 7.**
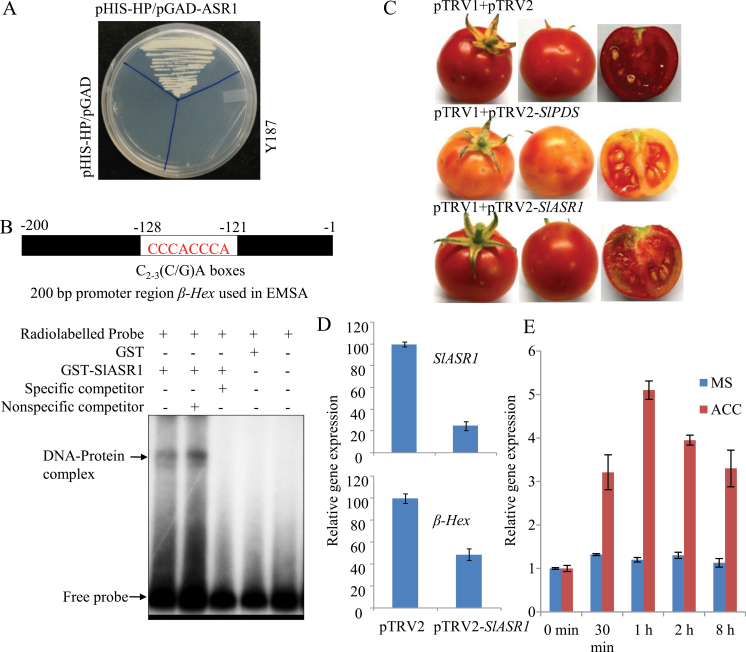
Transcriptional regulation of *β-Hex* expression by *SlASR1.* (A) Identification of SlASR1 as *β-Hex* promoter-interacting protein in Y1H screening. *β-Hex* promoter (HP) was inserted upstream of *HIS3* reporter in pHIS2.1 vector and co-transformed into *Saccharomyces cerevisiae* strain Y187 along with pGADT7 vector that carried complete *SlASR1* ORF upstream of the GAL4 transcription activation domain. Transformants were grown on SD medium lacking Trp, Leu, and His, but containing 5mM 3AT to determine *HIS3* reporter gene activation. Empty pGADT7 vector co-transformed with pHIS2.1-HP and untransformed strain served as the controls. (B) EMSA to confirm the interaction of SlASR1 with the *β-Hex* promoter. GST-tagged SlASR1 and a radiolabelled 200-bp sequence of *β-Hex* promoter were used for EMSA. GST served as a control. The specificity of the DNA–protein complex was determined by using an excess of specific (unlabelled probe) and nonspecific competitor (tomato actin sequence) DNAs. (C) VIGS of *SlASR1* in tomato fruits. For the silencing of *SlASR1, Agrobacterium* cultures containing pTRV1 and pTRV2-*SlASR1* vectors were mixed in 1:1 ratio and infiltrated into tomato fruits while attached to the plants. pTRV2 was used as a vector control and pTRV2-*SlPDS* was used as a VIGS positive control for silencing of *PDS*. (D) The transcript levels of *SlASR1* and *β-Hex* were determined in *SlASR1* silenced fruits through qRT PCR analysis. (E) *SlASR1* transcript level was determined in ACC-treated tomato seedlings by qRT-PCR analysis. Data are presented as the mean (±SE) of two biological replicates.

**Fig. 8.**
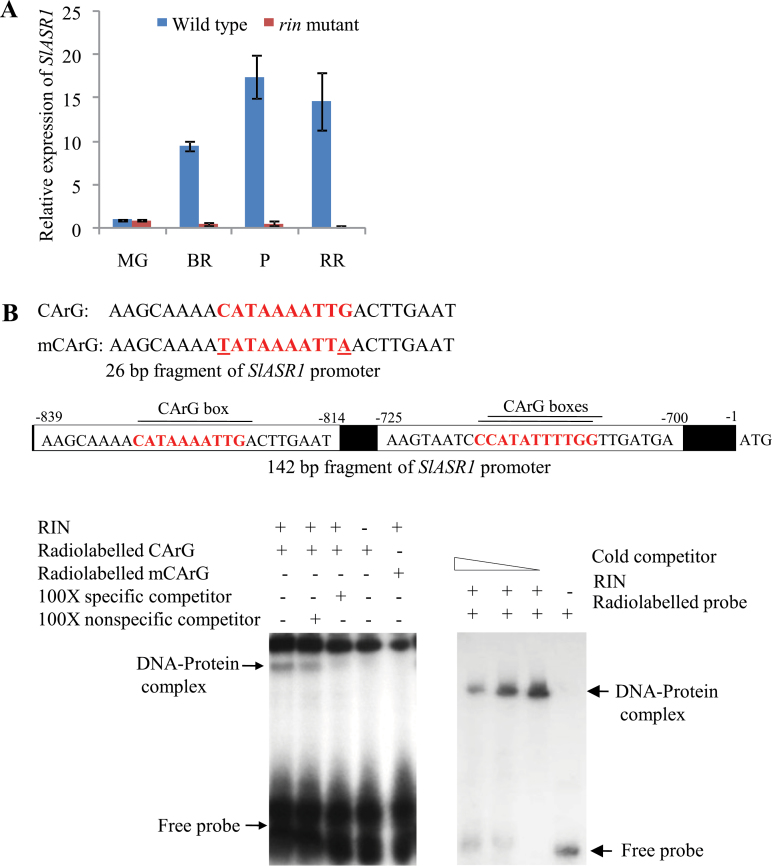
RIN regulates the expression of *SlASR1* during fruit ripening. (A) qRT PCR transcript analysis of *SlASR1* during ripening stages of wild type (cv. Ailsa Craig) and corresponding ages of *rin* mutant fruits. Data are presented as the mean (±SE) of at least three biological replicates. (B) EMSA to determine the interaction of RIN with the promoter fragments of *SlASR1.* Radiolabelled double stranded 26-bp (left side gel image) and 142-bp (right side gel image) fragments of *SlASR1* promoter were used as probes in EMSA. The specificity of the interaction was checked by using mutated CArG box (mCArG) and an excess of specific competitor DNAs (unlabelled probes) in EMSA. This figure is available in colour at *JXB* online.

In order to substantiate the result of the Y1H assay (Fig. 7A), the ability of SlASR1 to interact with the *β-Hex* promoter was confirmed through EMSA using bacterially expressed recombinant protein, GST-SlASR1 (Fig. 7B and Supplementary Fig. S5C). A part of the *β-Hex* promoter (–1 to –200bp) which contains a tandem repeat of the putative SlASR1 binding site [C_2–3_(C/G)A] was used as a probe in EMSA, which revealed specific interaction of SlASR1 with the *β-Hex* promoter sequence. GST alone did not show any binding activity. Although, the excess unlabelled 200-bp promoter DNA fragment was able to compete for the binding activity of GST-SlASR1, nonspecific DNA fragments (tomato actin sequence) were unable to compete (Fig. 7B). These results demonstrate specific binding of SlASR1 to the *β-Hex* promoter DNA fragment. Further, to confirm a role of *SlASR1* in regulation of *β-Hex* transcription *in planta*, VIGS-mediated suppression of *SlASR1* was carried out in tomato fruits using tobacco rattle virus (TRV)-based vectors (Liu *et al.*, 2002). A mixture of *Agrobacterium* suspension containing pTRV1 and pTRV2 vectors in 1:1 ratio was agroinjected into tomato fruits at 30 DAA to initiate VIGS (described in the materials and methods section). For silencing of *SlASR1,* pTRV1 and pTRV2-*SlASR1* (*SlASR1* cloned into pTRV2) vectors were used. However, the empty pTRV2 vector and pTRV2-*SlPDS* (tomato *phytoene desaturase* cloned into pTRV2) along with pTRV1 were used as controls in VIGS. Around 17 days after agroinjection, when pTRV1/pTRV2 vector-injected control fruits had progressed to the RR stage, pTRV1/pTRV2-*SlPDS* vector-injected fruits developed a yellowish phenotype due to low lycopene levels because of the suppression of *SlPDS*, confirming the efficacy of VIGS in tomato fruit (Fig. 7C). To test the silencing of *SlASR1* in VIGS fruits, the transcript level of *SlASR1* was determined through qRT PCR analysis (Fig. 7D). The analysis revealed up to 75% suppression of *SlASR1* expression in VIGS fruits. Interestingly, *β-Hex* expression was significantly downregulated (~50%) in *SlASR1* suppressed fruits as compared to the vector control (Fig. 7D). Moreover, *β-Hex* and *SlASR1* exhibited similar expression patterns during tomato fruit ripening, ACC treatment, and in ripening-impaired mutants (Figs 1, 4A, 6C, and 8A; and Supplementary Figure S7, at *JXB* online). Altogether, these results demonstrate that SlASR1 acts as a positive transcriptional regulator of *β-Hex* during fruit ripening.

ABA positively regulates the expression of *SlASR1* (Amitai-Zeigerson *et al.*, 1995). Moreover, the level of ABA in tomato fruits increases just prior to the induction of ethylene biosynthesis during fruit ripening, and depletion of ABA levels led to delayed ripening in tomato (Zhang *et al.*, 2009; Sun *et al.*, 2011 and 2012). Therefore, we tested whether *β-Hex* expression is also regulated by ABA. qRT-PCR expression analysis of *β-Hex* in wild type and *GUS* in HP::GUS transgenics after ABA treatment revealed upregulation of *β-Hex* and *GUS*, respectively (Fig. 4B and C). These results confirmed the role of ABA in positive regulation of *β-Hex* expression.

### Transcriptional regulation of *SlASR1* by RIN

RIN and SlASR1 directly interacted with the *β-Hex* promoter and the transcript level of *β-Hex* was down regulated when expression of either *RIN* or *SlASR1* was suppressed in tomato fruits (Figs 6A–D and 7B–D). Altogether, these results suggest that both RIN and SlASR1 are the positive transcriptional regulators of *β-Hex.* RIN is considered an upstream component of the ethylene-dependent and -independent fruit ripening pathways (Vrebalov *et al.*, 2002; Giovannoni, 2007; Fujisawa *et al.*, 2013). Moreover, *SlASR1* expression was upregulated during tomato ripening (Fig. 8A; Iusem *et al.*, 1993). Thus, we explored the possibility that *SlASR1* is under the transcriptional control of RIN. To address this issue, the expression level of *SlASR1* was assessed in *rin* mutants through qRT-PCR analysis, which revealed up to 90% suppression of *SlASR1* expression in *rin* mutants as compared to wild-type fruit (Fig. 8A). Further, to test whether RIN could directly interact with the *SlASR1* promoter, EMSA was carried out using a radiolabelled 26-bp *SlASR1* promoter DNA fragment with a CArG box element and its flanking sequences. We also used a radiolabelled 142bp region of the *SlASR1* promoter containing two overlapping CArG boxes for EMSA (Fig. 8B). These analyses revealed specific interaction of RIN with the *SlASR1* promoter sequence, suggesting a direct role for RIN in transcriptional regulation of *SlASR1*. Therefore, RIN could modulate the expression of *β-Hex* during fruit ripening, both directly and indirectly through SlASR1.

## Discussion

Although the physiological function of *β-Hex* in the ripening-associated fruit softening process has been elucidated (Jagadeesh and Prabha, 2002; Jagadeesh *et al.*, 2004; Meli *et al.*, 2010; Ghosh *et al.*, 2011; Cao *et al.*, 2014), the transcriptional regulation of *β-Hex* during fruit ripening is currently unknown. To gain an insight into the transcriptional regulation of *β-Hex* during fruit ripening, we have identified and functionally characterized the fruit ripening-specific promoter of *β-Hex*. The spatial and temporal expression patterns of the *GUS* reporter gene under the control of the *β-Hex* promoter were determined in transgenic tomato plants. Consistent with the fruit ripening-specific expression of *β-Hex* (Fig. 1), upregulation of promoter activity during tomato ripening was noticed (Fig. 2B–C). In fruits, maximum *β-Hex* promoter activation was recorded in pericarp tissues. However, *β-Hex* promoter was not activated in seedlings, roots, stems, leaves, and flower parts except sepals, which showed very low GUS activity (Fig. 3A–B). These results corroborate the role of *β-Hex* in ripening-associated fruit softening (Meli *et al.*, 2010; Ghosh *et al.*, 2011; Cao *et al.*, 2014). *In silico* analysis of the *β-Hex* promoter also revealed the presence of the *cis*-acting regulatory elements related to the fruit-ripening process, in addition to ubiquitous elements such as TATA and CAAT boxes (Supplementary Table S2). Moreover, a comparison of the expression profiles of *β-Hex* and *GUS*, in response to ACC and ABA treatment, in wild type and in HP::GUS transgenics, respectively, revealed similar patterns (Fig. 4A–C and Meli *et al.*, 2010). These results indicated that the 1001-bp *β-Hex* promoter region used to drive the *GUS* expression contains all the *cis*-acting regulatory elements indispensable for the spatio-temporal regulation of the endogenous *β-Hex* gene.

Our results suggest that MADS-box transcription factor RIN and SlASR1 are the positive regulators of *β-Hex* expression during fruit ripening. Both these proteins showed binding activities to the *β-Hex* promoter sequence (Figs 6A and B, and 7B) and *β-Hex* expression/promoter activity was decreased when the expression of either *RIN* or *SlASR1* was suppressed (Figs 6C and D, and 7C and D). *In silico* analysis of the *β-Hex* promoter also revealed the presence of CArG and [C_2–3_(C/G)A] boxes, the binding sites for RIN and SlASR1, respectively, thus, validating their role in transcriptional regulation of *β-Hex*. Interestingly, RIN was also found to act as a positive transcriptional regulator of *SlASR1*; because the expression of *SlASR1* was downregulated in *rin* mutant and RIN protein directly bound to the *SlASR1* promoter sequence that contains several CArG motifs (Fig. 8A and B). Thus, RIN may exert both direct and indirect control to positively regulate the expression of *β-Hex* during fruit ripening.

RIN is considered the global fruit ripening regulator (Vrebalov *et al.*, 2002). Previous studies have identified direct and indirect targets of RIN, including those involved in ethylene biosynthesis and signalling, fruit textural changes, and pigment biosynthesis (Ito *et al.*, 2008; Fujisawa *et al.*, 2011; Martel *et al.*, 2011; Fujisawa *et al.*, 2012; Kumar *et al.*, 2012; Qin *et al.*, 2012; Fujisawa *et al.*, 2013; Zhong *et al.*, 2013). Two earlier genome-wide chromatin immunoprecipitation experiments (ChIP-chip) revealed a list of genes including both *β-Hex* (Solyc01g081610.2.1) and *SlASR1* (Solyc04g071610.2.1) that are associated with the RIN binding sites (Fujisawa *et al.*, 2013; Zhong *et al.*, 2013); however, the precise role of RIN in regulating the expression of *β-Hex* and *SlASR1* during fruit ripening was not studied. Our results demonstrate that *β-Hex* and *SlASR1* are among the direct targets of RIN during fruit ripening. Conversely, the exact role of *SlASR1* in fruit ripening is yet to be determined. Although the nuclear localization of *SlASR1* has been fairly well investigated, its function as a transcriptional regulator is not well studied (Shkolnik and Bar-Zvi, 2008; Ricardi *et al.*, 2012). *SlASR1* showed induced expression during tomato ripening (Fig. 8A and Iusem *et al.*, 1993) and the hormones ethylene and ABA upregulated the expression of *SlASR1* (Fig. 7E and; Amitai-Zeigerson *et al.*, 1995). Both these hormones play crucial functions in tomato ripening (Zhang *et al.*, 2009; Sun *et al.*, 2011; Klee and Giovannoni, 2011; Sun *et al.*, 2012; Osorio *et al.*, 2013; Seymour *et al.*, 2013a, b). Moreover, the expression of *SlASR1* was downregulated in mutant fruits in which the expression of either of the fruit-ripening regulators *RIN*, *NOR*, and *NR* was suppressed (Fig. 8A and Supplementary Figure S7). In *nor* and *Nr* mutant fruits, the suppression of *SlASR1* expression may be due to the impaired biosynthesis and/or signalling of ethylene (Tigchelaar *et al.*, 1978; Wilkinson *et al.*, 1995; Vrebalov *et al*., 2002), because ethylene regulates the expression of *SlASR1* (Fig. 7F). Therefore, a role of *SlASR1* in tomato ripening cannot be ruled out. Earlier, ChIP-chip analysis using leaves from stressed tomato plants revealed several potential direct transcriptional targets of SlASR1; however, *β-Hex* was not identified (Ricardi *et al.*, 2014). Although, we do not exclude the possibility that some of the targets of SlASR1 are yet to be found, it is also likely that SlASR1 does not activate the *β-Hex* promoter in leaves. This is quite possible because *β-Hex* promoter remained inactive in leaves (Fig. 3A, B). Moreover, the methylation status of the promoter is likely to determine binding of the transcription factors to the promoter and further activation of *β-Hex* transcription. The *β-Hex* promoter region was found to exhibit differential methylation patterns in leaves and fruits during maturation and ripening (Zhong *et al.*, 2013). Interestingly, the differentially methylated region of the *β-Hex* promoter was hypomethylated during the progression of tomato ripening but hypermethylated in *rin* and another ripening mutant, *colorless non-ripening* (Zhong *et al.*, 2013).

Both ethylene and ABA positively regulate the expression of *β-Hex* (Fig. 4A–C and Meli *et al.*, 2010). *β-Hex* promoter was specifically activated in fruits during ripening (Fig. 2A–C) but remained inactive in seedlings, roots, stems, and leaves (Fig. 3A and B). Interestingly, *β-Hex* promoter-driven expression of *GUS* and *β-Hex* genes was noticed when seedlings (HP:GUS transgenics and wild type, respectively) were treated with ethylene and ABA (Fig. 4A–C). These results indicate that although *β-Hex* promoter remained inactive in normal seedlings, it is responsive to ethylene and ABA treatments. This also supports the essential roles of ethylene and ABA in activating the *β-Hex* promoter during natural fruit ripening, when the endogenous levels of these hormones increase. During ripening, the early induction of *β-Hex* at the BR stage might be brought about by ABA because the level of ABA in tomato fruit increases just prior to the P stage when ethylene attains the maximum level (Fig. 1; Meli *et al.*, 2010 and Sun *et al.*, 2012). Although *β-Hex* transcript level started increasing from the BR stage, maximum transcript level was attained at the P stage (Fig. 1; Meli *et al.*, 2010). Autocatalytic ethylene biosynthesis during the P stage might be responsible for high-level transcription of *β-Hex* at this stage. *SlASR1* expression is positively regulated by ethylene and ABA (Fig. 7E; Supplementary Figure S7 at *JXB* online; Amitai-Zeigerson *et al.*, 1995); thus, its involvement in both ethylene and ABA-regulated transcription of *β-Hex* cannot be excluded. Although ethylene is known to regulate *RIN* expression positively, the role of ABA is yet to be elucidated (Fujisawa *et al.*, 2013).

*β-Hex* exhibits similar expression patterns and plays a conserved physiological function during the ripening of both the climacteric fruit tomato, which requires ethylene for the ripening, and the non-climacteric fruit, capsicum, which does not require ethylene for ripening (Meli *et al.*, 2010; Ghosh *et al.*, 2011). In order to gain insight into the conserved transcriptional regulation of *β-Hex* during ripening of both climacteric and non-climacteric fruits, we tested capsicum *β-Hex* promoter-driven expression of *GUS* in tomato fruit (Fig. 5B). The activation of capsicum promoter in tomato fruits indicates that it contains all the essential *cis*-acting regulatory elements required for its activation in tomato. *In silico* analysis of tomato and capsicum *β-Hex* promoters revealed several common *cis*-acting regulatory elements including recognition sites for the MADS box transcription factor RIN and SlASR1, which could be involved in conserved transcriptional regulation of *β-Hex* in climacteric and non-climacteric fruits. Capsicum and tomato share the fruit ripening-regulatory components including the genes for ethylene biosynthesis and signalling; however, their regulation during fruit ripening differs in these fruits (Lee *et al.*, 2010; Osorio *et al.*, 2012; Kim *et al.*, 2014). Although the genes involved in ethylene biosynthesis do not exhibit induced expression during capsicum ripening, the expression pattern of the transcription factors (*RIN*, *TAGL1*, and *NOR*), ethylene signalling (*NR*, *ETR4*, *EIN2*, and *EIL*s), and downstream ethylene target genes, such as those involved in cell wall metabolism and fruit pigment biosynthesis, was conserved during fruit ripening. A similar expression pattern of RIN in these fruits and complementation of RIN function by the capsicum orthologue suggest its possible involvement in the transcriptional regulation of *β-Hex* during ripening of capsicum fruit as well (Kim *et al.*, 2014; Dong *et al.*, 2014). In conclusion, this work led to the isolation of the fruit ripening-specific promoter of *β-Hex* and identification of *RIN* and *SlASR1* as the transcriptional regulators of *β-Hex*. Together, the results demonstrate that RIN and SlASR1 directly bind to the *β-Hex* promoter and positively regulate the expression of *β-Hex* during tomato ripening.

## Supplementary material

Supplementary data can be found at *JXB* online.

Supplementary Table S1. List of primers used in the study.

Supplementary Table S2. The putative *cis*-acting regulatory elements identified within tomato and capsicum *β-Hex* promoters through *in-silico* analysis (NewPLACE, PlantCARE, and MatInspector).

Supplementary Figure S1. Genomic organization of tomato *β-Hex*.

Supplementary Figure S2. Tomato *β-Hex* promoter isolation by the PCR-based genome walking method.

Supplementary Figure S3. Isolation of the capsicum *β-Hex* gene promoter.

Supplementary Figure S4. Sequences of *β-Hex* and *SlASR1* promoters showing position of the CArG boxes.

Supplementary Figure S5. Purification of recombinant GST-RIN and GST-SlASR1 protein from *E. coli.*

Supplementary Figure S6. The mRNA expression of putative *β-Hex* promoter-binding protein genes.

Supplementary Figure S7. The mRNA expression of *SlASR1* in wild-type and ripening mutants *nor* and *Nr.*

## Funding

This work was financially supported by a research grant (BT/01/CEIB/12/II/01) from the Department of Biotechnology and core research grant from the National Institute of Plant Genome Research.

## Supplementary Material

Supplementary Data
