# Supplementary material for: Insights into transcriptional regulation of β-D-N-acetylhexosaminidase, an N-glycan-processing enzyme involved in ripening-associated fruit softening
Source: J Exp Bot. 2014 Aug 16;65(20):5835–48. doi: 10.1093/jxb/eru324 (PMC4203122; doi:10.1093/jxb/eru324)
Supplement: Supplementary Data [file supp_65_20_5835__index.html]

Insights into transcriptional regulation of β-D-N-acetylhexosaminidase, an N-glycan-processing enzyme involved in ripening-associated fruit softening — Insights into transcriptional regulation of β-D-N-acetylhexosaminidase, an N-glycan-processing enzyme involved in ripening-associated fruit softening — Supplementary Data 

# Insights into transcriptional regulation of β-D-N-acetylhexosaminidase, an N-glycan-processing enzyme involved in ripening-associated fruit softening

## Supplementary Data

Data files

**Files in this Data Supplement:**

- Supplementary Data - Supplementary Data
