# Supplementary material for: Insights into transcriptional regulation of β-D-N-acetylhexosaminidase, an N-glycan-processing enzyme involved in ripening-associated fruit softening
Source: J Exp Bot. 2014 Aug 16;65(20):5835–48. doi: 10.1093/jxb/eru324 (PMC4203122; doi:10.1093/jxb/eru324)
Supplement: Supplementary Data [file supp_eru324_jexbot126904_file001.pdf]

SUPPLEMENTAL DATA

Insights into transcriptional regulation of β-D-N-acetylhexosaminidase, an N-glycan processing enzyme involved in ripening-associated fruit softening

Mohammad Irfan, Sumit Ghosh, Vinay Kumar, Niranjan Chakraborty, Subhra Chakraborty and Asis Datta

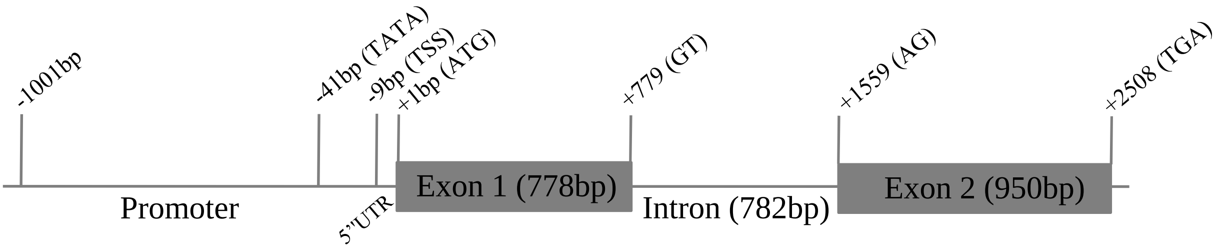

Figure S1. Genomic organization of tomato *β-Hex*. Putative transcription start site (TSS) is based on rapid amplification of cDNA ends (5'RACE, Clontech) and TATA box is based on NewPLACE (https://sogo.dna.affrc.go.jp) and PlantCARE (bioinformatics.psb.ugent.be) analysis.

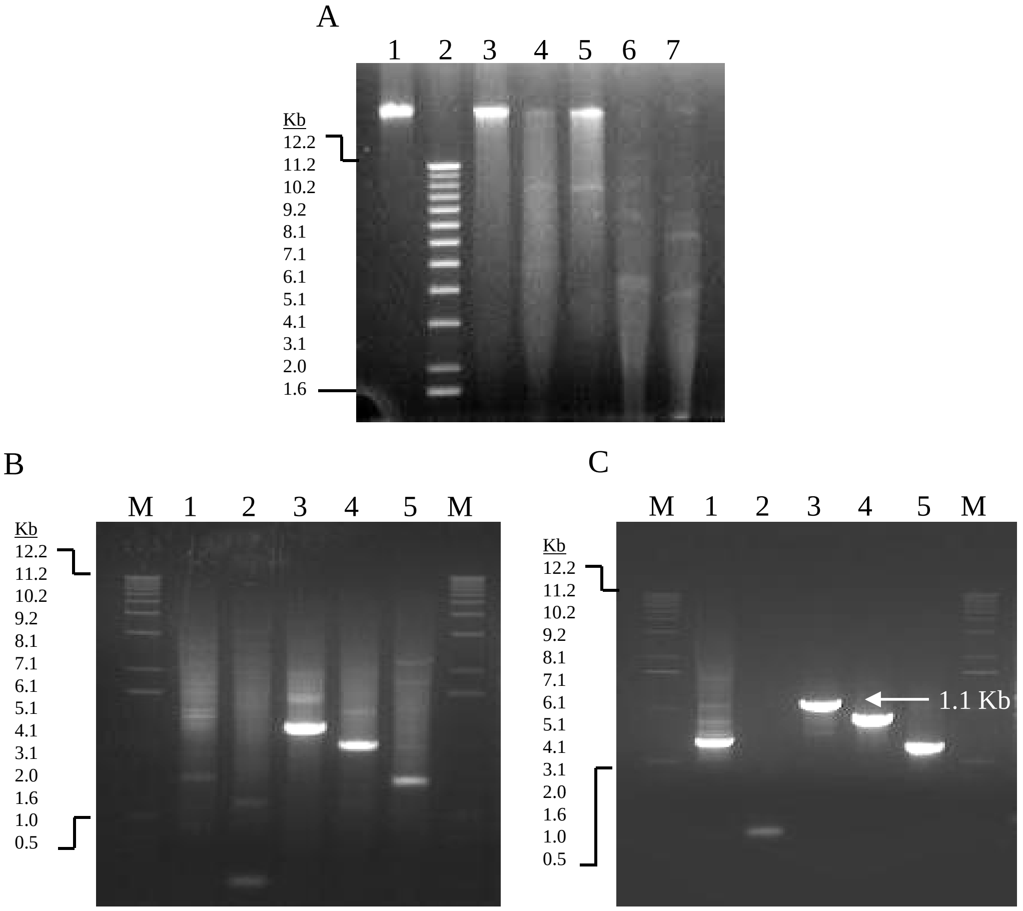

Figure S2. Tomato *β-Hex* promoter isolation by PCR based genome walking method. (A) Digestion of tomato genomic DNA with *Pvu*II (lane 3), *Xmn*I (lane 4), *Msc*I (lane 5), *Dra*I (lane 6) and *Ssp*I (lane 7). Lane 1- undigested DNA and Lane 2- 1 Kb ladders (Invitrogen). Genome Walker Adapters (Clontech) were ligated to the digested DNA fragments and were referred as libraries. (B) Primary PCR with adaptor-specific (AP1) and gene-specific (GSP1) primers using the libraries constructed as mentioned in A. Lane 1-5 *Pvu*II, *Xmn*I, *Msc*I, *Dra*I and *Ssp*I libraries. (C) Secondary PCR with the AP2 and GSP2 primers using diluted primary PCR product as the template. The arrow indicates 1.1 kb fragment amplified from *Mse*I library, which was cloned into pGEM-T easy vector and sequenced.

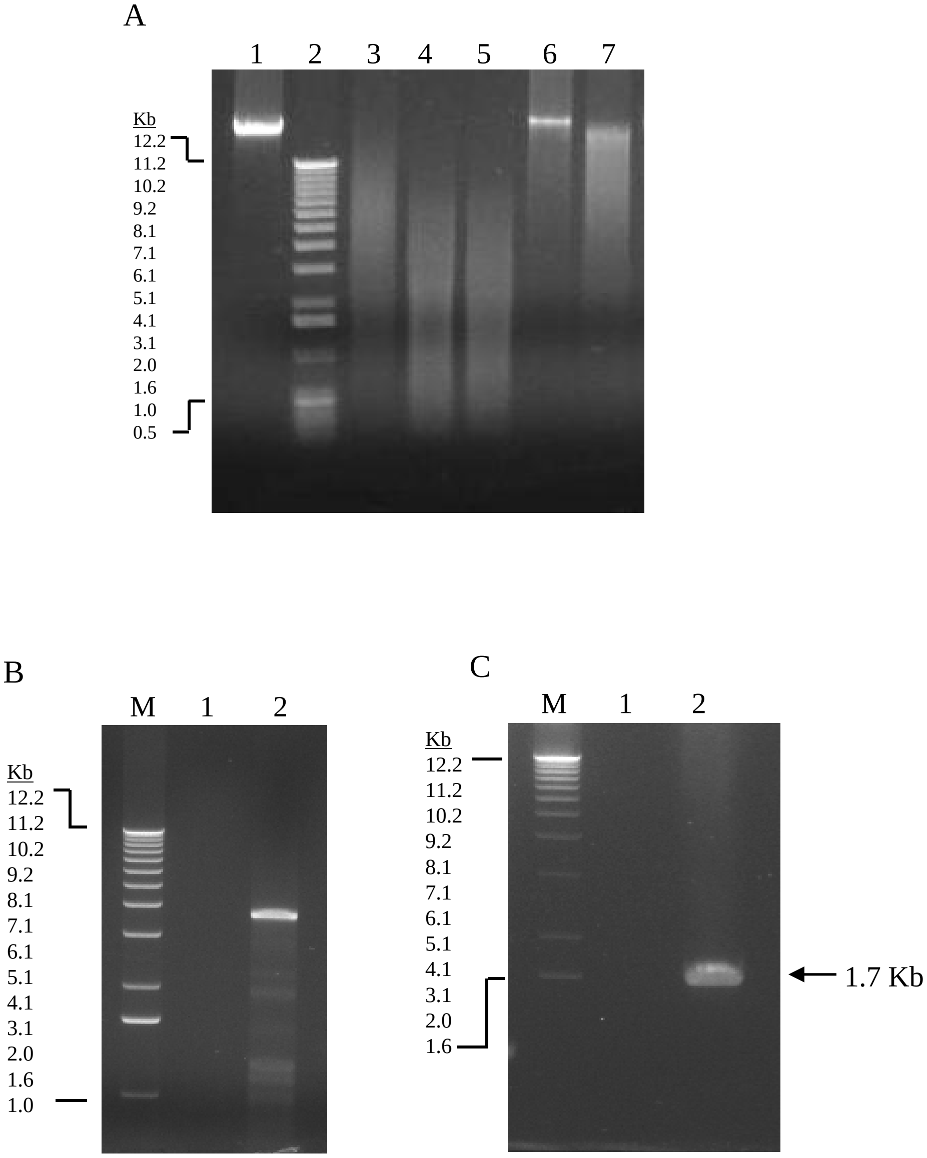

Figure S3. Isolation of capsicum *β-Hex* gene promoter. (A) Digestion of capsicum genomic DNA with *Xmn*I (lane 3), *Dra*I (lane4), *Ssp*I (lane 5), *Msc*I (lane 6) and *Sma*I (lane 7). Lane 1- undigested genomic DNA and lane 2- 1 Kb ladders (Invitrogen). Genome Walker Adapters (Clontech) were ligated to the digested DNA fragments and were referred as libraries. (B) Primary PCR with adaptor-specific (AP1) and gene-specific (GSP1) primers using the libraries constructed as mentioned in A. PCR amplification was attained only in *Dra*I library (lane 2). (C) Secondary PCR with the AP2 and GSP2 primers using diluted primary PCR product as the template. The arrow indicates 1.7 kb fragment amplified from *Dra*I library, which was cloned into pGEM-T easy vector and sequenced.

Tomato *β-Hex* promoter:

-1001 -992
CCAATTTAGGGGTCAATGACTATTTTTAAATCTATATTCCTCTCGCCTGCAGTCTCTTTAAGCTTTGTCTCACTAATCACAACTA
CArG Box 1

-900 -891 -870 -861
ATGAGTTGTTTATAAA**CTTATAAAAG**T**TGGG**TTAAATGAGTTGTAT**CATTTAATTG**TTTTTTATATATTTAAAATTAGGTGAAA
CArG Box 2 ASR1 binding site CArG Box 3

-818 -809
ATTTTTTCTTATCT**CATTTAAAAG**TTAATTCTGATTCATGCTACTTAAAGCTAAACTTACCAACATAATAAGCACAAATAACCAA
CArG Box 4

-720 -711
TGAATATTTGTCTAAACTTCACAAAAA**CATAATTATG**CTTCTTACTTTTATAATTCAAGAAAAGTTCAAATTGTTTATTTAGGAA
CArG Box 5

AGAAGAGAAGAAACAAAAAGAAAAAAACAAAAAAAAAAAAAACAAAGAAGAACAAATGACTCAAAGTTATAATAATGTATG
GATCACTTGAAAATTTATTCGCTAAAGGCCTATAGTCGTTTGGTAGCTGGTTTAGATGCAATCTTGTATAAGTAATATTATGTTT
GCTAGTTAACTAGAAAATAAGTATTAAATTAATACAGTGTTTGATTTGTAATTTAGAAATTCGAATAACTAATACATGCACACG
TTAAAAAGAAAATCCAAGTATCATTTATGCAGGACAAGAGATAGAATAACTGATAAATGTATCATTTATTAATCACTCCATTAC
TAATATCTGCATAAAGTAATGAGTACATACATTTAGTCATAACTTTAACCAACTACTAAACGACACTTAAAAGGATAATAAAAA
CATTAAGTTTAAGTTGCTTGTATCCAAATCACAAACCAACAAATTAGAAGCAAAGACTTTTGAAGTTGAAAAGAAAGTAGCCA

-128 -125 -121
AGTCAAGTCTTTATACAATCCCCTTTCATCCAT**CCCACCCA**AAATATAAACTCCAAACATAATCAAACAGAACAATGTAATTTT
ASR1 binding sites

-71 -62 **-1**
CATATT**CCAAAATTTG**ATATTTTAGAAAAATAACAATATAAATAGTCCATGTAATTTTCATTTCTCTTGAGAAAAAA
CArG Box 6

Capsicum *β-Hex* promoter:

-883 -879
TATTCTTTTAATAAAATATTACTACAATTTATATTTAGTTGAACTTTTCTTTTTACTAAAAAAAAAATA**CCCA**ACTTTATATCGTCA
ASR1 binding site

GAATATATAATTCACAATTGTGATGAACGACTATTTCTAACCTTTAGCTGTTGGCCTGCAATCTCTCTAAACTAATCATTTTAAG

-737 -728
TCATCATTCCTTTTAGTTTACTTCTTTTAGTTTTTTTTTTCTC**CATTAAATTG**ATTTTTTGGATTTCATTTTATTTGCATTGGTAAAT
CArG Box 1

-669 -666
AGTATCAATGGAATTACCTTTG**TGGG**CTGTGGATCAACTTTTGTTTCATGTCTCTAATTTGTCTCACCAATCATGACTCATATGA
ASR1 binding site

-527 -523
GTTGTGTATAAACATAAAAGTTAGGTTAAATACCCTGACTTATTATTTACTGCTATTGGTTAAGTGCATCTAGTCAATAA**CCGA**A
ASR1 binding site

-470 -467
AAGCAAAGTACTTAGTCAATACGAGAAACTAAACGTAAACGCTTTTCTTTAT**CCCA**TCTAAAAGGTAATGCTAATTCATGCTTA
ASR1 binding site

-373 -364
AGCTAGATTTACCAACATTATAAGCACAAAATTACAATGAATATTTGTCTAAACTATCATTTTCA**CTTAATTTAG**TGTGCAGCA
CArG Box 2

AGGATAAATTTGGAAAAAATATTCTGCTAAACATTAAAGTTTCAGTCACTTGATAATCCAAATCACAAACCAACAAATTAGAAG
CAAAGACTTTTAAATCTAAAAATAAAGTAAACATGTCAAGTCTTTATATATAGTCCTTCCATCCATCCATTCAATCCAAACACTA

-112 -103
TCAAACAAACTACCTAGCTATTTCCAAAGTAATATCACAAGATTCAACAAACAAACAATACTCTTATCATATT**CCAAAATTTG**A
CArG Box 3

-42 -38 -29 -26
TATTTAGAAAATCAACATAATAAATAGTGCTTTCTTCGTATTTGTTTTTCTTCAAACCC**CCCCA**ATCCGCTC**TCGG**TCGTGACTA
CTAACGGGTCATCAAA
ASR1 binding site ASR1 binding site

SlASR1 promoter:

CACGTAACAAAAATATATATATCTCAGTGTAGAATACATAAAAAAAATTTTAATTAGTGATAAAATATATAATATATTAAAAAT
ATAAATAATAATAATATATATAATAATAAAGTATGTCTAATTAGGTAGTTTTTCTTTTTGAAAACTGAAATGAGAAAAAGCAAA

-822 -831
A**CATAAAATTG**ACTTGAATGACAGCTACATGACATTTTCATCTTGTAGTAGGGACATATGATTTGTTTTTTTCCTTTGCCACATG
CArG Box 1

-717 -708
-716 -707
TGTTCTGTTATCCTTAATCTCCAAGTAATC**CCATATTTTGG**TTGATGATTCACAATATAATCTATCTAATTATGCACCTCCTTCT
CArG Box 2&3

ACTTAAAGAAGAAAAATGTGATGGCGATTGGCAATTGGGAAGATAATTAAAATCTGTTGAGTACTCTTTCATCCGCAATGGCAT
TCAGTCGATGGAACAATAGTGAAAGAGATGTTTAAAAAAATTATTTACATTTAAAATGATTTTAGATTTGACGCAATCCGAAAA
AATTAGTCTATAAAAAAAAATTATTTAAAATCATGCAAGAGCTCAATTAACTTCATCCGCCTTTGATGTGAGTTTTTCTACATTCA
TCACGCTTCCCATCCCCGAACCCCAACACTCTATACTCCGATCCATGACGTGAACAAATTATTCAAGCGTTCAATTTGACTCTAA
TATCATACTAAATAAACCTAATTTAATAGTAAAAATTAGCTTAACAATTTACTAATTTCACACAATTTTTTATATTGTTGTCTTGT
CATTATCTTTAGGTAATAATAGTGTAAAAATTATCTTACACGATTATACTACATAATTTATACGATTCGTTGATAAATTGTATAC
CAAAGTGCCACCTCATCACACAATAATTTAATTTGGACTAAGTTCACTATTAGTGAATGAATGAATTTTAATTATAAATAGAGG
ACTTGACAAGATCATATTTGTATCAAACACCATACACTTTCTAAATTATCGATAGATTTATTGTTTCAG

Figure S4. Sequences of *β-Hex* and *SlASR1* promoters showing position of the CArG boxes. Putative SlASR1 binding sites are also depicted within the *β-Hex* promoters. Differential methylation regions within the tomato *β-Hex* promoter as determined by Zhong *et al.*, 2013 is highlighted with red colour.

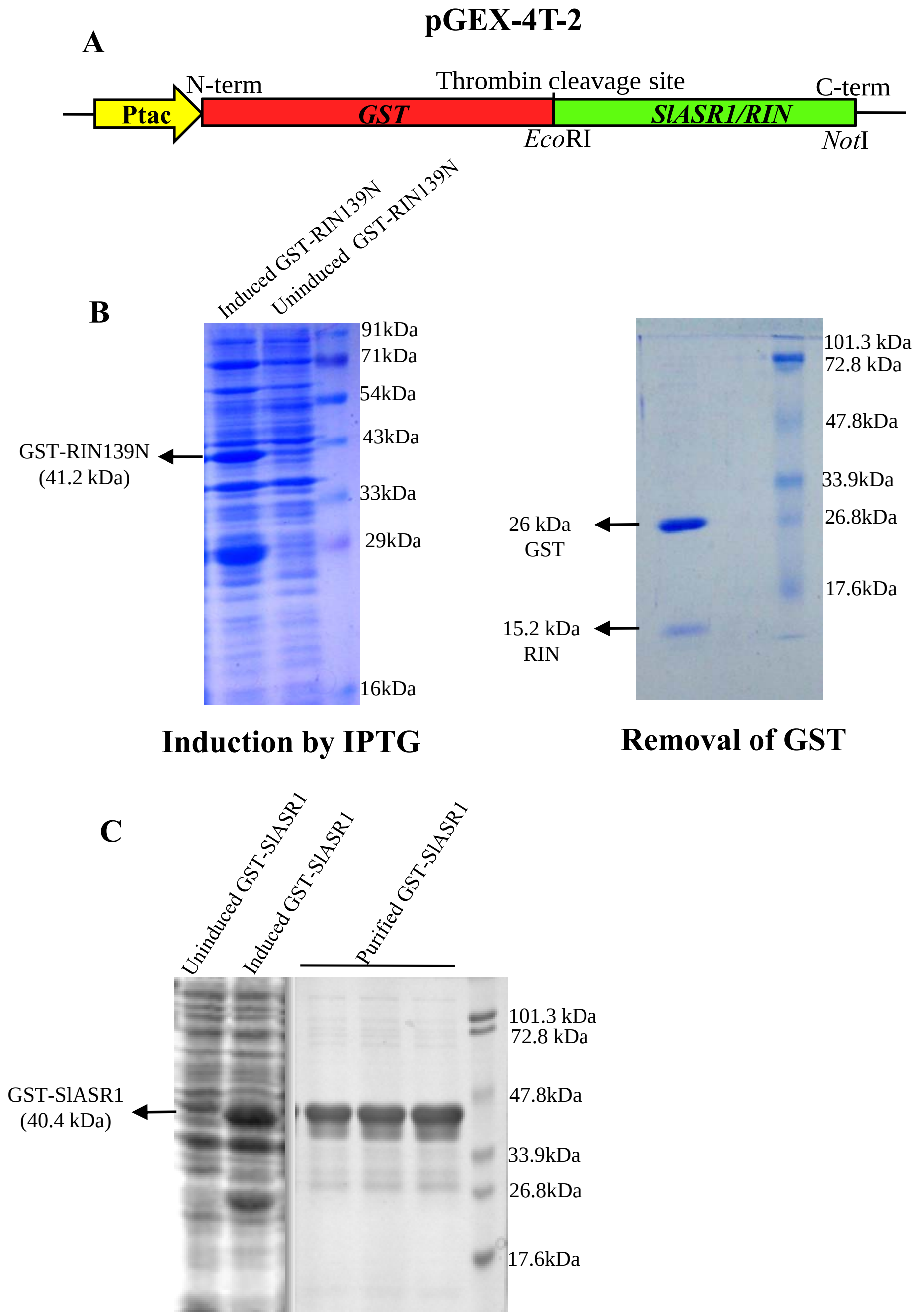

Figure S5. Purification of recombinant GST-RIN and GST-SlASR1 protein from *E. coli.* (A) Full length coding region of *SlASR1* and N-terminal 139aa of RIN (RIN139N) were cloned in pGEX4T-2 expression vector in frame to N-terminal GST tag and transformed into *E. coli* BL21 cells. Maximum induction of GST-SlASR1 and GST-RIN139N was found at 28°C with 0.6 mM IPTG. Induced proteins were purified by affinity chromatography by using Glutathione Sepharose 4B matrix and analyzed on 12.5% SDS-PAGE. (B, C) The GST-SlASR1 and GST-RIN139N proteins were resolved as 40.4 kDa and 41.2 kDa polypeptides, respectively on SDS-PAGE. The GST tag from GST-RIN139N protein was removed by using thrombin. These 15.2 kDa RIN139N and 40.4 kDa GST-SlASR1 proteins were used for EMSA.

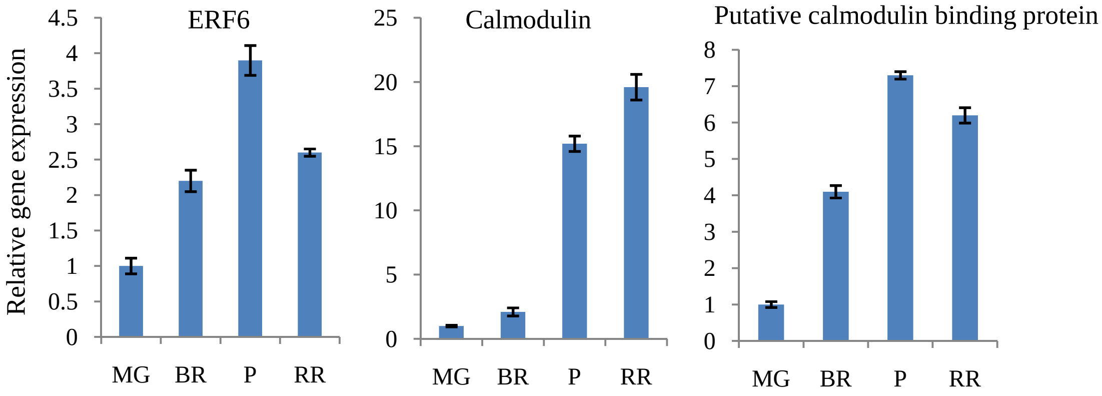

Figure S6. The mRNA expression of putative *β-Hex* promoter binding protein genes (ERF6, Calmodulin and putative Calmodulin binding protein) was determined through qRT-PCR at different ripening stages. Data are presented as the mean (±SE) of at least three biological replicates.

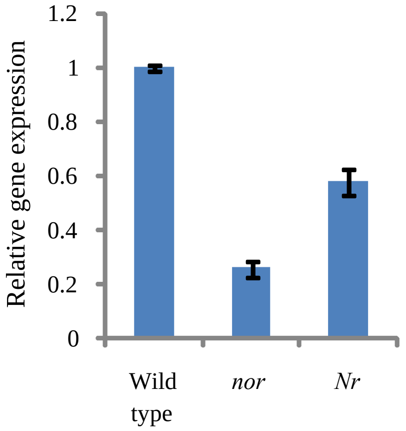

Figure S7. The mRNA expression of *SlASR1* in wild type (cv. ailsa craig) and ripening mutants *nor* and *Nr*. Data are presented as the mean (±SE) of two biological replicates.

Table S1. List of primers used in the study

Primer sequences	Purpose
HPF1: CCCAAGCTTCTCGAGGTCTCACTAATCACAACTAATGAG<br>HPF2: CCGCTCGAGCCAATTTAGGGGTCAATGACT<br>HPR: GCTCTAGATTTTTTCTCAAGAGAAATGAA	Preparation of tomato *β-hex* promoter::*GUS* fusion construct.
HPD4: CCCAAGCTTTAGAAGCAAAGACTTTTGAAG TTG<br>HPR: GCTCTAGATTTTTTCTCAAGAGAAATGAA	Isolation of 200 bp region of *β-hex* promoter (probe used for EMSA).
CapHPF: CCCAAGCTTTATTCTTTTAATAAAATATTACTACAATTTATATTTAG<br>CapHPR: GCTCTAGATTTGATGACCCGTTAGTAGTCAC	Preparation of capsicum *β-hex* promoter::*GUS* fusion construct.
RTTAL: TTATCACCATTGGTGCTGAG<br>RTTAR: CGATGTTTCCATACAGATCCTT	qRT-PCR of tomato *actin* gene as endogenous control.
RTH1F: TATGTTCTGGTGGCCCG<br>RTH1R: TCTGCTCCTCCGTGAAAG	qRT-PCR of tomato *β-hex* gene.
RTGUSF: CGGCAAAGTGTGGGTCAATA<br>RTGUSR: GCAATAACATACGGCGTGACA	qRT-PCR analysis of *GUS* gene.
PGXASF2: GGAATTCTGGCCGGGGGTATCAAACACCAT<br>PGXASR: ATTTGCGGCCGCTTAGAAGAGATGGTGGTGTCCC	Cloning of *SlASR1*in pGEX 4T2 vector.
RTASRF: CATGGAGGCCAGTGAATCTCA<br>RTASRR: CCCCCGGCCATAATGG	qRT-PCR of tomato *SlASR1* gene.
ASRTF: CGGAATTCATGGAGGAGGAGAAACACCA<br>ASRTR: GCTCTAGAGAAGAGATGGTGGTGTCCCC	Cloning of *SlASR1* in pTRV2.
pGEX-RINF: GGAATTCTGGGTAGAGGGAAAGTAGAATT<br>pGEX-RINTR: ATTTGCGGCCGCTCACCTAATTTGCCTCAATGAT	Cloning of *RIN* in pGEX 4T2 vector
RTRINF1: TAGTCGTGGCAAGCTTTATGAATT<br>RTRINR: TGTATCTGTGGTATCTCTCCAATGTCT	qRT-PCR of tomato *RIN* gene.

Table S2. The putative cis-acting regulatory elements identified within tomato and capsicum *β-Hex* promoters through *in-silico* analysis (NewPLACE, PlantCARE and MatInspector).

Transcription factor	Tomato *β-Hex* promoter		Capsicum *β-Hex* promoter	
	Sequence	Position	Sequence	Position
Ethylene insensitive 3 (EIN3) like factors, involved in ethylene regulated gene expression.	aTGTAtgta	697 – 705 (-)	______	______
MADS box protein (RIN)	CCAATTTAGG<br>CTTATAAAAG<br>CATTTAATTG<br>CATTTAAAAG<br>CATAATTATG<br>CCAAAATTTG	1-10 (+)<br>102-111 (+)<br>132-141 (+)<br>184-193 (+)<br>282-291 (+)<br>931-940 (+)	CATTAAATTG<br>CTTAATTTAG<br>CCAAAATTTG	215-224 (+)<br>579-588 (+)<br>840-849 (+)
Abscisic Acid Stress Ripening protein 1	CCCA<br>CCCA<br>CCCA	878-881 (+)<br>874-877 (+)<br>113-116 (-)	CCCA<br>CCCCA<br>CCCA<br>CCGA<br>CCCA<br>CCGA<br>CCCA	911-914 (+)<br>910-914 (+)<br>482-485 (+)<br>425-428 (+)<br>69-72 (+)<br>923-926 (-)<br>282-285 (-)
Calcium regulated NAC-factors/ Calmodulin binding NAC protein (CNAC)	ttgtGCTTattatgttggtaa<br>agttGCTTgtatccaaatcac	226 – 246 (-)<br>769 – 789 (+)	ctttGCTTttcggttattgac<br>tctaGCTTaagcatgaattag<br>ttgtGCTTataatgttggtaa	417 – 437 (-)<br>500 – 520 (-)<br>522 – 542 (-)
Auxin response element (AREF)	cttTGTCtcacta	63 – 75 (+)	attTGTCtcacca	316 – 328 (+)
Brassinosteroid response element (BRRE)	tttaaCGTGtgcatgta	578 – 594 (-)	______	______
ABA response elements (ABRE)	ctttttaACGTgtgcat	581 – 597 (-)	______	______
MYB-like proteins (MYBL)	ttaaatgAGTTgtatca<br>aaaaaacaATTAaatga<br>gtttgcTAGTtaactag<br>atttatcaGTTAttcta<br>tttatattGTTAttttt<br>AtgtatTAGTtattcga<br>agtagtTGGTtaaagtt	117 – 133 (+)<br>131 – 147 (-)<br>502 – 518 (+)<br>631 – 647 (-)<br>950 – 966 (-)<br>566-582 (-)<br>714-730 (-)	tatattTAGTtgaactt<br>tcatatgAGTTgtgtat<br>gcttttcgGTTAttgac<br>gaaaagcgTTTAcgttt<br>cttataatGTTGgtaaa<br>ggtagtTTGTttgatag<br>gctaggTAGTttgtttg<br>gtttgtTTGTtgaatct<br>gatgacccGTTAgtagt	30 – 46 (+)<br>337 – 353 (+)<br>417 – 433 (-)<br>460 – 476 (-)<br>521 – 537 (-)<br>764 – 780 (-)<br>768 – 784 (-)<br>806 – 822 (-)<br>932 – 948 (-)
Sucrose box (SUCB). Found in sucrose responsive	ctAAATaaacaatttgaac	318 – 336 (-)	aaAAATcaatttaatggag	212 – 230 (-)

genes.	acAAATcaaacactgtatt<br>taAAAAcattaagtttaag<br>aaATATcaaattttggaat	536 – 554 (-)<br>752 – 770 (+)<br>928 – 946 (-)	atAAGTcagggtatttaac<br>taATATcacaagattcaac<br>aaATATcaaattttggaat<br>gaAAATcaacataataaat	369 – 387 (-)<br>796 – 814 (+)<br>837 – 855 (-)<br>857 – 875 (+)
W Box family	AgtcaTTGAcccctaaa	5-21 (-)	ggttaTTGActagatgc<br>tcgtaTTGActaagtac	410-426 (-)<br>437-453 (-)
Regulatory element involved in the MeJA-responsiveness	______	______	CGTCA<br>TGACG	81 (+)<br>81 (+)
Cis-acting element involved in gibberellin-responsiveness (GARE-motif)	AAACAGA	904 (+)	TATCCCA	478 (+)
Cis-acting element involved in defense and stress responsiveness	______	______	ATTTTCTCCA	206 (+), 894 (+)<br>607 (-)
Fungal elicitor responsive element (Box-W1)	TTGACC	10 (-)	______	______
Cis-acting element involved in salicylic acid responsiveness (TCA element)	GAGAAGAATA	34 (-)		
ARR1AT, "ARR1-binding element" found in Arabidopsis; ARR1 is a response DE regulator (Ross *et al*., 2004).	NGATT	201 (+), 547 (+)<br>29 (-), 75 (-)<br>480 (-), 600 (-)<br>660 (-), 784 (-)<br>857 (-), 901 (-)	NGATT	518 (+), 806 (+)<br>232 (+), 223 (+)<br>146 (-), 160 (-)<br>328 (-), 652 (-)<br>658 (-), 694 (-)
DOFCOREZM, core site required for binding of Dof proteins Dof proteins are DNA binding proteins, with presumably only one zinc finger, and are unique to plants; Yanagisawa, 2000	AAAG	108 (+), 190 (+)<br>216 (+), 315 (+)<br>315 (+), 356 (+)<br>383 (+), 402 (+)	AAAG	361 (+), 429 (+)<br>434 (+), 490 (+)<br>633 (+), 683 (+)<br>705 (+), 792 (+)
GATABOX, required for light regulated gene expression. Benfey *et al.*, 1990	GATA	629 (+), 641 (+)<br>747 (+), 940 (+)<br>129 (-), 179 (-)	GATA	600 (+), 649 (+)<br>849 (+), 79 (-)<br>262 (-), 479 (-)
GT1CONSENSUS, found in promoter of light regulated genes. Zhou, 1999	GRWAAW	166 (+), 359 (+)<br>429 (+), 518 (+)<br>597 (+), 641 (+)<br>747 (+), 949 (+)	GRWAAW	253 (+), 493 (+)<br>600 (+), 609 (+)<br>610 (+), 649 (+)<br>857 (+), 272 (-)
GT1CORE, light responsive element	GGTTAA	115 (+),718 (-)	GGTTAA	368 (+), 402 (+)
IBOXCORE	GATAA	641 (+), 747 (+)<br>178 (-)	GATAA	600 (+), 649 (+)<br>478 (-), 830 (-)
